# Darier’s disease exhibits a unique cutaneous microbial dysbiosis associated with inflammation and body malodour

**DOI:** 10.1186/s40168-023-01587-x

**Published:** 2023-07-26

**Authors:** Yacine Amar, Danielle Rogner, Rafaela L. Silva, Bärbel U. Foesel, Minhaz Ud-Dean, Ilias Lagkouvardos, Susanne A. Steimle-Grauer, Sebastian Niedermeier, Susanne Kublik, Manja Jargosch, Matthias Heinig, Jenny Thomas, Stefanie Eyerich, Jakob D. Wikström, Michael Schloter, Kilian Eyerich, Tilo Biedermann, Martin Köberle

**Affiliations:** 1 Department of Dermatology and Allergy, Technical University of Munich, School of Medicine, Munich, Germany; 2 Research Unit Comparative Microbiome Analysis, Helmholtz Zentrum München, Deutsches Forschungszentrum Für Gesundheit Und Umwelt (GmbH), 85764 Neuherberg, Germany; 3 Institute of Computational Biology, Helmholtz Zentrum München, German Research Center for Environmental Health, 85764 Neuherberg, Germany; 4 Department of Informatics, Technical University of Munich, Garching, Germany; 5 Core Facility Microbiome, Technical University of Munich, 85354 Freising, Germany; 6 Center of Allergy & Environment (ZAUM), Technical University of Munich (TUM) and Helmholtz Zentrum München, German Research Center for Environmental Health, Member of the German Center of Lung Research (DZL), Munich, Germany; 7 Dermatology and Venereology Division, Department of Medicine Solna, Karolinska Institutet, Stockholm, Sweden; 8 Dermato-Venereology Clinic, Karolinska University Hospital, Stockholm, Sweden; 9 Department of Dermatology and Venereology, Medical Center, University of Freiburg, Freiburg, Germany

**Keywords:** Genodermatosis, Darier’s disease, Skin barrier, Malodour, Microbiome, Transcriptome

## Abstract

**Background:**

Darier’s disease (DD) is a genodermatosis caused by mutations of the *ATP2A2* gene leading to disrupted keratinocyte adhesion. Recurrent episodes of skin inflammation and infections with a typical malodour in DD indicate a role for microbial dysbiosis. Here, for the first time, we investigated the DD skin microbiome using a metabarcoding approach of 115 skin swabs from 14 patients and 14 healthy volunteers. Furthermore, we analyzed its changes in the context of DD malodour and the cutaneous DD transcriptome.

**Results:**

We identified a disease-specific cutaneous microbiome with a loss of microbial diversity and of potentially beneficial commensals. Expansion of inflammation-associated microbes such as *Staphylococcus aureus* and *Staphylococcus warneri* strongly correlated with disease severity. DD dysbiosis was further characterized by abundant species belonging to *Corynebacteria*, *Staphylococci* and *Streptococci* groups displaying strong associations with malodour intensity. Transcriptome analyses showed marked upregulation of epidermal repair, inflammatory and immune defence pathways reflecting epithelial and immune response mechanisms to DD dysbiotic microbiome. In contrast, barrier genes including claudin-4 and cadherin-4 were downregulated.

**Conclusions:**

These findings allow a better understanding of Darier exacerbations, highlighting the role of cutaneous dysbiosis in DD inflammation and associated malodour. Our data also suggest potential biomarkers and targets of intervention for DD.

Video Abstract

**Supplementary Information:**

The online version contains supplementary material available at 10.1186/s40168-023-01587-x.

## Background

**Fig. 1:**
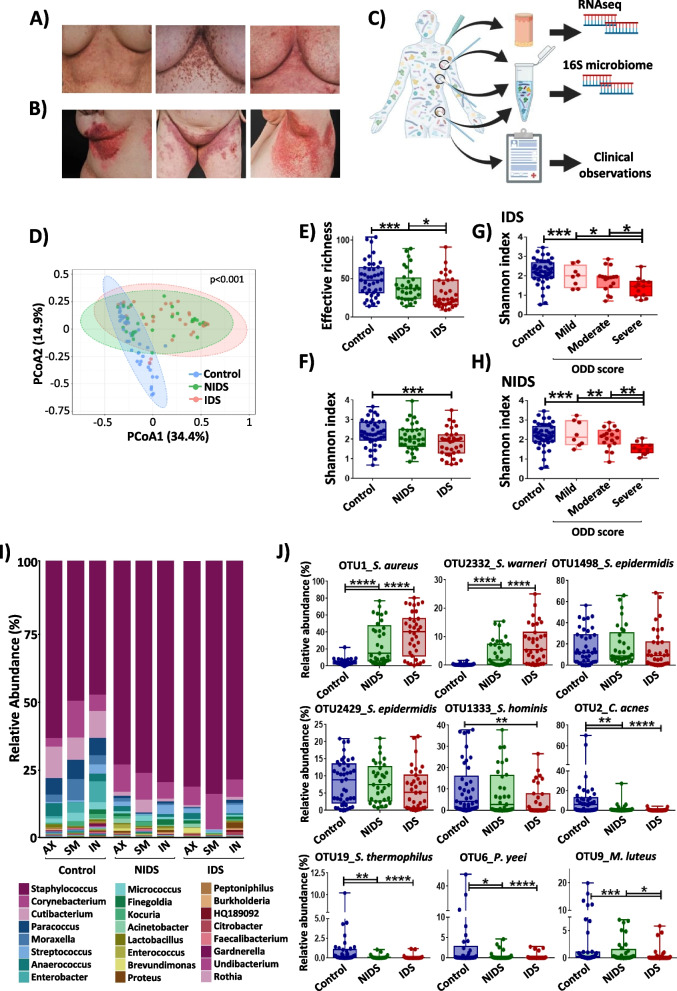
Cutaneous microbiome in DD is characterized by a reduced α-diversity and strong dysbiotic shifts. **A** Representative pictures for submammary skin of Darier’ patients with mild (ODD: 16.5), moderate (ODD: 29) and severe (ODD: 50.5) objective DD scores (from left to right). **B** Erythematous and hyperkeratotic papules observed on DD predilection areas. **C** Workflow of microbiome and transcriptome sampling and analysis. Samples were collected from axillary (AX), submammary (SM) and inguinal (IN) areas. Created with BioRender.com. **D** Principal coordinate analysis (PcoA) plot of β-diversity of microbiota from healthy, non-inflamed (NIDS) and inflamed (IDS) DD skin microbiomes. The Bray–Curtis index was used to calculate the similarity between samples and PERMANOVA to test the statistical significance based on the distance matrix. **E**, **F** α-diversity expressed as effective richness (number of OTUs) and the Shannon index are shown for control healthy skin, NIDS and IDS. Shannon index displayed according to the objective DD score for **G** IDS and **H** NIDS skin compared to control samples. **I** Bar chart of taxonomy binning displayed at the genus level. The taxonomic composition was assessed by summing up OTUs relative abundances that share the same assignment at the genus level. The Bayesian classifier from the RDP database was used for OTUs classification. **J** Relative abundances plots of dominant taxa *S. aureus*, *S. warneri*,* S. epidermidis*,* S. hominis*,* C. acnes*,* S. thermophilus*,* P. yeei* and* M. luteus*. Each dot represents a swab sample. Multiple test corrections were performed with the Benjamini and Hochberg procedures. The statistical significance was calculated using Kruskal–Wallis and Wilcoxon-Mann–Whitney tests, respectively, for multiple group and pairwise comparisons. The asterisks indicate statistically significant differences and correspond to *p ≤ 0.05, **p ≤ 0.01, ***p ≤ 0.001, and ****p ≤ 0.0001

Darier’s disease (DD) is a rare autosomal dominant genodermatosis, first reported by Darier [1] and White [2] in 1889. DD prevalence ranges between 1/30000 (Scotland) [3] and 1/100000 (Denmark) [4] with no gender preference and an estimated new cases incidence of 4 per million per 10 years [5]. DD skin is characterized by hyperkeratotic red-brown papules with scaly plaques predominantly appearing on seborrheic skin areas (chest, intertriginous, forehead, scalp margins and the back), usually fading into or with relatively adjacent non-lesional skin areas (Fig. 1a, b) [6]. In the human gene mutation database, 302 mutations of the *ATP2A2* gene encoding the sarco/endoplasmic reticulum Ca^2+^ ATPase isoform 2 (*SERCA2*) are listed for DD [7]. The loss of function of *SERCA2* leads to abnormal intracellular Ca^2+^ signalling. This causes both acantholysis (loss of intercellular adhesion) and anoikosis (a form of programmed cell death), which as a result leads to dyskeratosis. In histology, dyskeratosis manifests as corps ronds (large apoptotic keratinocytes surrounded by a clear halo) and grains (compressed cells with elongated nuclei) [8]. DD follows a chronic course with fluctuating severity including exacerbations of skin inflammation and recurrent cutaneous infections. Several triggering factors for these exacerbations have been identified, including humidity, heat, excessive sweating, pregnancy, exposure to UV light and mechanical irritation [9]. Among the factors of burden in DD are skin inflammation and the main symptoms of pruritus and malodour, observed in 88% or 44% of patients, respectively, also leading to substantial social isolation [5]. Several findings indicate that measures to prevent or inhibit inflammation in DD will alleviate the disease burden, which is a highly unmet need, because of the severely impaired quality of life for these patients [10].

Importantly and in contrast to other inflammatory skin diseases, DD inflammation is not a primary consequence of the mutation. Indeed, in approximately 70% of patients, the disease is first noticed between the age of 6 and 20 years, with a peak during puberty (11–15 years of age) [5], when also the skin microbiota is re-shaped [11]. Increased susceptibility to bacterial, viral and fungal infections is well documented in DD. They are generally localized and associated with cutaneous inflammation, but can also be life-threatening or fatal in some cases [12, 13]. Underlying mechanisms explaining this high susceptibility to cutaneous infection are believed to be based on skin barrier defects in DD. There has been, however, some evidence for impairments in Langerhans cells, plasmacytoid dendritic cells or B cells [14, 15]. From other inflammatory skin diseases such as atopic dermatitis (AD), we know that microbial dysbiosis is tightly connected to disease pathogenesis, associated to barrier defects and a driver of disease pathogenesis especially of inflammation [16–20]. However, detailed analyses of DD microbiome have not been performed so far and possible links of dysbiosis to inflammation in DD are also not established. Based on the mentioned disease characteristics, defining changes in DD skin microbiome promises to deliver new insights into DD pathogenesis, among them specific pathways of inflammation. In addition, DD is clearly defined by the loss of function of *SERCA2* and not based on a complex genetic trait as many other inflammatory skin diseases. It may thus serve as a good model to better understand the general crosstalk between the cutaneous microbiome and the host’s inflamed skin. Therefore, a national German registry for DD patients was founded (https://bit.ly/3efDRuM) to better characterize and study DD patients and to possibly develop new strategies for intervention. Here, we present for the first time data from microbiome and transcriptome analysis in DD involving patients of this registry and show how a mutation of a single gene in DD shapes skin microbiome and inflammation. These findings offer a comprehensive insight into DD cutaneous microbial composition and identify key microbial drivers of inflammation and the associated skin signatures underlying DD exacerbations.

## Methods

### Study design

All participants in the study were selected according to the inclusion/exclusion criteria approved by the regional government of Oberbayern. Medical ethical committee approval (565/18 s) and individual written informed consent were obtained in advance of any sample collection. Fourteen Darier patients were recruited at the dermatology hospital of the Technical University of Munich and followed up during regular visits all along the course of this study. Furthermore, as there is no validated scoring approach for DD, patients were examined using a scoring method based on SCORAD (Scoring of Atopic Dermatitis) [21] (Supplementary methods, Fig. S1). The proposed score uses similar items to assess the objective DD score (ODD Score) including severity (hyperkeratosis and erosions) and extent (surface of affected skin areas) scores. The global DD score (DD Score) is calculated by summing up ODD and the subjective score (pain and pruritus ranging from 0 to 10). To reduce inter-individual variety, the same dermatologist performed all the scoring. While future validation will be necessary to integrate it into the daily routine, the DD score is a helpful tool for assessing disease severity in patients and despite the limitations and the subjectivity, it sheds light on the disease characteristics in a more detailed manner. Furthermore, as there is no validated score for DD yet, we used the physician’s global assessment commonly performed in psoriasis to classify disease severity into clear (0), almost clear (1), mild (2), moderate (3) and severe (4) [22]. According to the dermatologist also assessing the ODD score, the patients were classified into mild (PGA 2; 4 cases), moderate (PGA 3; 6 cases) and severe (PGA 4, 4 cases). The PGA evaluation was well aligned with the established ODD scoring (Table 1), and the latter was therefore used for participants’ categorization into severity groups of “mild” (ODD-score: < 20), “moderate” (ODD-score: 20–35) and “severe” (ODD scores: > 35). Patients’ odour has also been scored on a scale from zero (not perceptible) to ten (very intense) by an experienced dermatologist [23], as this is one of the predominant symptoms in DD. The investigator categorized the patients into mild (score ≤ 3), moderate (score 4–6) and intense (score ≥ 7) odour groups (Table 1). Sample collection guidelines included in the Human Microbiome Project [24] were used (see details in supplementary methods).

**Table 1:** Clinical characteristics of Darier’s patients included in the cohort

**ID**	**Age**	**Gender**	**Therapy at sampling**	**Age at onset**	**ODD score**	**DD score**	**Odour score**	**PGA**
**DD1**	78	Female	Acitretin	19	27 ++	40	6^oo^	Moderate (3)
**DD2**	58	Female	None	14	37.5 +++	51.5	7^ooo^	Severe (4)
**DD3**	56	Female	LDN	15	50.5 +++	58.5	9^ooo^	Severe (4)
**DD4**	30	Female	LDN	19	16.5 +	31.5	1^o^	Mild (2)
**DD5**	55	Female	None	15	19 +	25	4^oo^	Mild (2)
**DD6**	45	Female	Laser	19	7 +	9	1^o^	Mild (2)
**DD7**	48	Female	None	25	29 ++	35	5^oo^	Moderate (3)
**DD8**	29	Male	Acitretin	13	59 +++	75	8^ooo^	Severe (4)
**DD9**	48	Male	Acitretin, ISTR	8	80 +++	96	10^ooo^	Severe (4)
**DD10**	58	Male	None	28	28 ++	33	3^o^	Moderate (3)
**DD11**	25	Male	Acitretin	19	23 ++	28	1^o^	Moderate (3)
**DD12**	52	Female	Acitretin	21	24 ++	30	1^o^	Moderate (2)
**DD13**	29	Male	Acitretin	14	24 ++	30	5^oo^	Moderate (3)
**DD14**	27	Male	None	15	20.5 +	25.5	2^o^	Mild (2)

*ODD* objective Darier’ disease score, *DD* Darier’ disease score, *PGA* physician global assessment, *LDN* low-dose naltrexone, *ISTR* isotretinoin, *ODD score-based severity groups* mild + , moderate ++ , severe +++ , *Odour score-based groups*: mild ^o^, moderate ^oo^ and intense ^ooo^; participant’s IDs were ordered according to the date of involvement in the study

### Specimen collection

Skin swabs were collected from inflamed (IDS) and non-inflamed (NIDS) submammary, inguinal and axillar regions of Darier patients and corresponding sites on their healthy matched controls. The NIDS was defined as the adjacent unaffected skin areas at a distance of 5 to 10 cm from the IDS, as in classical DD the inflamed areas are in transition to non-lesional skin areas. Control swabs were maintained in the air for 20 s, then similarly processed to exclude swab’s contaminating reads. Microbial DNA was extracted from freshly collected samples using a benzonase pre-digest approach that we optimized to better assess the living fraction of the skin microbiota [25]. DNA samples were stored at − 30 °C until further processing.

Skin biopsies were collected by the recruiting dermatologist from ten patients, including 10 IDS and 9 NIDS samples. No biopsies were collected from the healthy matched controls. Samples were divided into two parts, one collected in paraformaldehyde for histological analysis and the second part dedicated to transcriptomics was stored in RNAlater at − 80 °C. The total RNA was isolated from bulk biopsies using the miRNeasy Mini Kit according to the manufacturer’s protocol. Purified RNA samples were stored at − 80 °C until further use (see details in supplementary methods).

### 16S rRNA gene amplification and downstream processing

The 16S rRNA gene metabarcoding targeting the V3-V4 regions was used in this study to explore the microbial diversity. A composite pool was prepared by combining 4 nM of purified amplicon samples ensuring equal representations of barcoded libraries sequenced on an Illumina MiSeq platform with a PE300 v3 cartridge generating up to 25 million of 2 × 300 bp reads. The obtained reads have been analysed following the UPARSE method as implemented in the online IMNGS platform [26]. Briefly, primer and barcode sequences were trimmed from each read, and sequences shorter than 200 bp, low-quality and chimeras were discarded. The cleaned sequences were clustered into OTUs based on a similarity cutoff of 97% and taxonomically classified with the RDB classifier. Downstream analysis including diversity, taxonomy binning, serial group comparison and correlations was performed on R program (R software 4.0.2) using R scripts available in the Rhea pipeline [27]. The non-parametric Kruskal–Wallis rank sum test was used for multiple group comparison, and the Mann–Whitney test when only two groups were compared. Multiple test corrections were performed with the Benjamini and Hochberg procedure and corrected *P* values of less than 0.05 considered as significant (see details in supplementary methods).

### RNAseq library preparation and downstream analysis

RNASeq libraries were generated using the TruSeq Stranded Total RNA Kit (Illumina) according to the manufacturer’s protocol and sequenced on an Illumina HiSeq4000 as paired-end with a read length of 2 × 150 bp and an average output of 40 Mio reads per sample and end. STAR aligner was used to perform sequence alignment with the human reference genome hg38. Short- and low-quality reads were discarded, and the obtained clean reads were processed using the DESeq2 package for differential gene expression analysis (FCH ≥ 1.5, adj. *p* < 0.05 and FDR < 0.05). Gene set enrichment analysis has been performed using the GSEA 4.1.0 platform through which DD transcriptomes were analysed for enrichment in different gene set clusters implemented in databases including KEGG, GOBP (gene ontology for the biological process), HP (human phenotype) and PID (pathway interaction database). A network was designed for the most differentially expressed genes between IDS and NIDS skin transcriptomes using Cytoscape 3. Gene clusters were identified with ClusterViz using the MCODE algorithm (molecular complex detection) and their number was determined with the elbow method (see details in supplementary methods).

## Results

### Study population

Patients with DD (*n* = 14) and healthy matched (age, gender) controls (*n* = 14) were included in this study (Table 1; Table S1). DD patients were characterized in detail with regard to DD presentation including clinical signs (erythematous, warty and hyperkeratotic papules, plaques, erosions), affected areas (size and location) and the course of their disease. Figure 1A shows representative pictures of DD submammary areas with mild, moderate and severe intensities from left to right. Skin inflammation was most frequently seen on the axillary (AX), submammary (SM) and inguinal (IN) areas (Fig. 1B), previously reported as predilections sites in DD [5]. These observed lesions were all categorized as inflamed Darier skin (IDS) while the non-inflamed Darier skin (NIDS) was defined as the adjacent unaffected skin. As no DD-specific clinical scoring system has been evaluated so far, we established a DD score and an ODD score that is based on objectively measurable parameters only (Fig. S1). The mean ODD score for all patients was 31 ± 18 (mean ± SD), and DD score was 40 ± 21 (mean ± SD). The PGA evaluation was well aligned with this newly established ODD scoring (Table 1), and the latter was therefore used for participants’ separation into severity groups. In addition, a detailed medical history was documented for each patient.

All DD patients included in this study reported a disease onset at puberty (average age: 17.4 ± 4.9 years) as previously shown for most DD patients [5]. Eleven (79%) participants reported on at least one episode of cutaneous bacterial infection in the 2 years prior to inclusion into the study and on anti-bacterial treatment(s) either systemically (clindamycin or cefuroxime) or topically (antiseptic wash, clindamycin or creams containing fusidic acid). Ten (71%) DD patients reported on DD exacerbations due to cutaneous infections, of which nine (64%) had been hospitalized due to disease severity within these two years. Eight patients (57%) had received systemic therapy for DD in the past, seven of those (50%) retinoids (isotretinoin and/or acitretin), two (14%) low-dose naltrexone (LDN), one (7%) both LDN and a retinoid. At the time of sampling, six patients (43%) received retinoids, two (14%) LDN and the other five (35%) had no systemic treatment (Table 1). The most common symptoms/complaints reported were pruritus (ten patients: 71%), malodour (nine patients: 64%) and pain on erosive skin areas (four patients: 36%).

### Darier’s skin microbiome is characterized by a pronounced dysbiosis and reduced microbial diversity

In order to determine the consequences of *SERCA2* haploinsufficiency on DD cutaneous microbiome, skin swabs were collected from defined skin areas (AX, SM, IN) of IDS and NIDS as well as swabs from healthy matched controls. In addition, skin biopsies were taken from DD patients at the affected areas (Fig. 1C). A total of 115 swabs were collected and analysed yielding in a total of 3.27 × 10^6^ filtered reads with an average number of high-quality reads of 2.84 × 10^4^ per sample, resulting in 348 different OTUs (operational taxonomic units) overall analysed samples. Five samples showed low read counts, below 2000 reads, and were therefore excluded from the analysis. We first analysed β-diversity, a distance-based ecological measure for group comparison using principal coordinate analysis (PCoA). Results of β-diversity indicated a distinct composition of bacteria in DD skin samples compared to healthy controls with no marked differences between IDS lesions and rather asymptomatic NIDS (Fig. 1D, Fig. S2A-C). However, bacterial α-diversity expressed as richness or Shannon index showed intermediate values for NIDS, which ranged between control and IDS groups with a significant decrease for IDS compared to healthy controls (Fig. 1E, F). This indicates that the microbiome of DD at non-inflamed skin areas still preserves putatively healthy microbiota despite the overall similarity with IDS. Importantly, the drop of Shannon diversity was more pronounced for IDS (Fig. 1G) and NIDS (Fig. 1H) from severely affected patients than for “moderate” and “mild” groups compared to healthy controls. These disparities persisted when skin microbiomes from predilection sites were analysed separately (Fig. S2D, E), indicating that the clinical attributes of “severe” and “inflamed” are associated with more pronounced shifts of the cutaneous microbiome. Importantly, although a number of patients still received retinoid-based therapy (isotretinoin and/or acitretin) or low-dose naltrexone (Table 1), this treatment did not exert a significant impact on the microbiome composition of the IDS and NIDS skin (Fig. S3A, B) and has been the least contributing factor to the batch effect (Fig. S3C). However, it could not be fully excluded that these treatments would also affect the skin microbiome composition which deserves to be investigated in a larger cohort.

Next, we examined the relative abundance of skin taxa to learn more about the bacteria thriving on DD skin and potentially contributing to DD exacerbations. Microbiome analysis of AX, SM and IN skin of healthy individuals showed at a prevalence cut-off of 0.25% and abundance cutoff of 0.5: 52, 48 and 60 genera, respectively. *Staphylococcus*, *Corynebacterium* and *Cutibacterium* were detected in all healthy IN samples and in more than 93% of SM and AX swabs. Further typical commensals like *Paracoccus*, *Streptococcus*, *Moraxella* and *Micrococcus* species were also present in most samples from healthy participants (Fig. 1I). In DD, all sites investigated showed increased proportions of Staphylococci on NIDS and IDS that were paralleled by reduced fractions of *Cutibacteria* (Fig. 1I, Fig. S4A-C). Analysis at the species level (97% similarity of OTUs) showed high relative abundances of reads linked to *Staphylococcus aureus* and *Staphylococcus warneri* from DD skin samples with a concomitant drop of *Staphylococcus epidermidis*, *Staphylococcus hominis*, *Cutibacterium acnes*, *Streptococcus thermophilus*, *Paracoccus yeei* and *Micrococcus luteus*, all commonly categorized as potentially beneficial skin commensals (Fig. 1J). *S. aureus* was the common *Staphylococcus* species obtained from DD skin samples being detected in 93% of the samples derived from affected skin areas. The corresponding reads displayed a relative abundance of 40% on SM-IDS and IN-IDS and about 33% on the AX-IDS. These reads were also increased on NIDS with 30%, 24% and 32%, respectively, on AX, SM and IN sites (Fig. S5A-D). *C. acnes* was present in 81% of healthy skin samples with a mean relative abundance of 10% that was significantly reduced to 1% in samples from DD skin. *S. hominis* proportions were reduced by half on affected SM and AX locations, whereas *S. epidermidis* tended to decrease only slightly. The abundance of *Paracoccus yeei* was noticeably reduced in DD dropping from 6%, 3.7% and 3.3% in healthy AX, SM and IN samples to 0.14%, 0.01% and 0.65% on respective inflamed IDS areas. Similar observations were made for *Micrococcus luteus* and *Moraxella osloensis* in samples obtained from affected DD skin (Table S2). Overall, samples from non-inflamed DD skin showed intermediate proportions of both potentially pathogenic and commensal taxa when compared to IDS and control groups. This indicates that NIDS areas still conserve features of a healthy balanced skin microbiome. In conclusion, DD skin displayed specific changes of the microbiota with an overabundance of potential pathogens like *S. aureus* and *S. warneri* at the cost of potentially beneficial commensal species and skin microbial diversity.

### Key members of Darier’s cutaneous microbiome strongly correlate with disease status

**Fig. 2:**
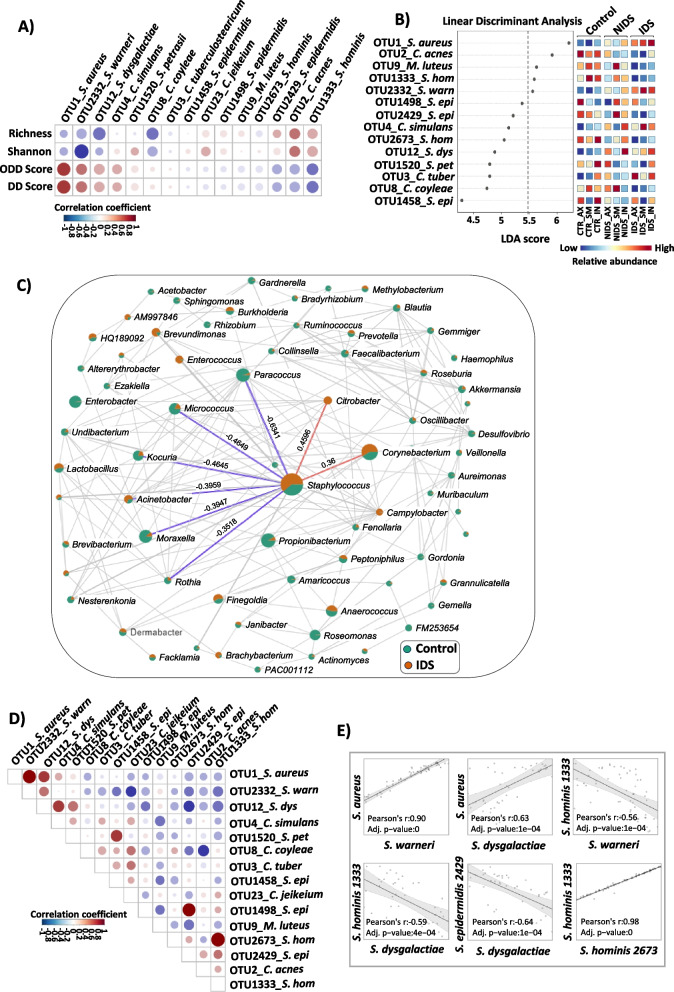
Key members of DD cutaneous microbiome correlate with disease severity and may serve as microbial disease markers. **A** Correlation analysis of key taxa with α-diversity (richness and Shannon index), DD score (global severity score) and ODD score (objective severity score). **B** Linear discriminant analysis effect size (LEFSe) of key taxa distribution over the different skin locations. LEFSe employs the Kruskal–Wallis rank sum test to detect OTUs with significant differential abundances between groups (control, NIDS and IDS), the pairwise Wilcoxon test between sub-groups, followed by a linear discriminant analysis (LDA) to evaluate the relevance or effect size of each differentially abundant taxon. The heatmap to the right shows in which group the taxa relative abundance is increased and the LDA score displays the potential marker taxa of the group. We considered in our analysis that the strongest associations between key taxa and skin phenotype are best delineated above the LDA cutoff of 5.5. Red and blue colors on the LDA plot indicate high and low taxa relative abundances, respectively. **C** Correlation network of microbiome communities in IDS and control groups displayed at the genus level. The SparCC (sparse correlations for compositional data) approach has been used to define network associations. This approach assumes a sparse network and performs iterations to identify taxa pairs that are outliers to background correlations. Each node represents a taxon and its size is proportional to the number of connections. The green and orange colors on the nodes indicate the taxa mean relative abundance in the control and IDS groups, respectively. Taxa are only connected if the correlation meets a *p* value cutoff of 0.05 and a correlation coefficient of 0.3. Key correlations with the *Staphylococcus* genus are highlighted in the figure with blue and red lines, representing negative and positive correlations, respectively. **D** Correlation analysis of key taxa relevant in DD pathology. **E** Linear correlation plots of representative taxa interactions. Pearson’s coefficient was used to calculate correlations among taxa or between taxa and metavariables. Red and blue colours on the correlation plots respectively indicate positive and negative correlations. Statistical significance was calculated using Kruskal–Wallis and Wilcoxon-Mann–Whitney tests, respectively, for multiple group and pairwise comparisons. The asterisks indicate statistically significant differences and correspond to *p ≤ 0.05, **p ≤ 0.01, and ***p ≤ 0.001

To evaluate whether the relative abundances of the key identified taxa are associated with DD severity assessed by our proposed scores, we performed a correlation analysis. To this end, we selected the 15 most regulated OTUs and compared their occurrence with microbial richness, Shannon index, ODD and DD severity scores (Fig. 2A; in red positive, in blue negative correlations). Obtained data revealed that *S. aureus*, *S. warneri* and *S. dysgalactiae* relative abundances were negatively correlated with microbial diversity. Moreover, these OTUs showed positive correlations with DD as well as the ODD severity scores (Fig. 2A). In contrast, putatively beneficial commensals including *S. epidermidis*, *S. hominis* and *C. acnes* displayed the opposite trend and were rather linked to a balanced microbiome as they displayed positive and negative correlations respectively with α-diversity and disease severity (Fig. 2A). We next performed a linear discriminant analysis effect size (LEFSe) to evaluate the association between the identified key taxa and disease status, to assess their utility as DD biomarkers. The linear discriminant analysis (LDA) plot in Fig. 2B displays a linear representation of OTU predictors showing significant differential abundances between control, IDS and NIDS groups. Importantly, strong correlations between key taxa and the skin phenotype are indicated by high LDA scores, in our study best above an arbitrary cutoff of 5.5 (heatmap to the right with relative abundance high in red; Fig. 2B). Thus, *S. aureus* and *S. warneri* appear as predictive markers for the DD group displaying high LDA scores of 6.21 and 5.56, respectively, whereas the healthy skin microbiome was rather defined by potentially beneficial commensals including *C. acnes*, *M. luteus* and *S. hominis*, all with elevated LDA scores above the arbitrary cutoff of 5.5.

To better characterize potential interactions between members of the bacterial community in the context of a dysbiotic DD, we investigated the correlations between key taxa regulated in DD microbiome. Network analysis of taxa correlation at the genus level, both for “NIDS vs control” and “IDS vs control” groups, revealed that Staphylococci are negatively correlated with several genera including *Micrococcus*, *Moraxella*, *Kocuria*, *Paracoccus* and *Acinetobacter*, of which many members are considered as beneficial skin commensals (Fig. 2C, Fig. S6). In contrast, several other taxa, including *Corynebacteria* expanded together with Staphylococci*,* pointing towards either resilience to *Staphylococci*-induced suppression or even a potentially synergistic regulation of expansion. At the species level, the correlation analysis revealed a significant positive correlation between *S. aureus* and *S. warneri* pointing toward a synergistic regulation. Contrarily, *S. epidermidis*, *S. hominis*, *C. jeikeium* and *C. acnes* displayed negative correlations with both *S. aureus* and *S. warneri* indicating potential antagonistic regulations (Fig. 2D, E).

### Malodour in DD correlates with cutaneous microbiome dysbiosis

**Fig. 3:**
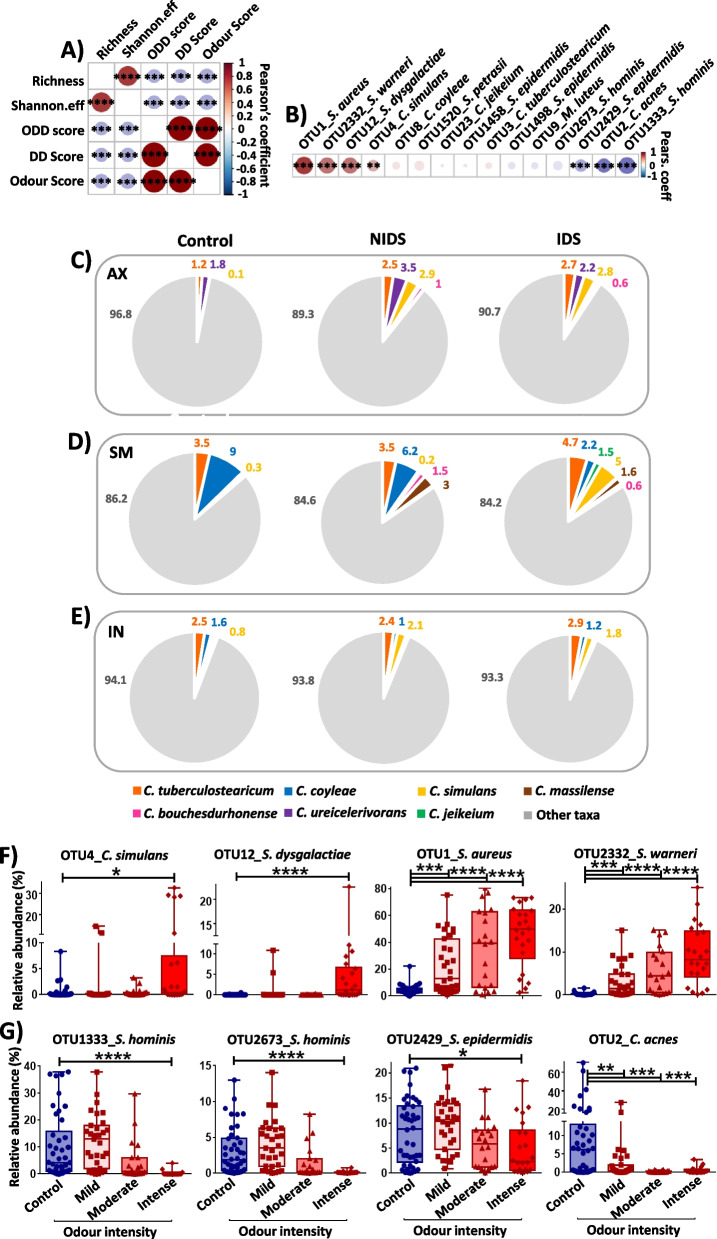
Skin dysbiosis is associated with malodour in DD. **A** Correlation plot of α-diversity, disease severity and malodour in Darier patients. **B** Association between key abundant taxa and DD malodour. Pearson’s coefficient was used to calculate correlations among metavariables or between taxa and odour intensity. Red and blue colors on the correlation plots indicate positive and negative correlations, respectively. **C** Pie charts of *Corynebacterium* taxa distribution on DD axillary **D** submammary and **E** inguinal locations. The *Corynebacterium* group shows a threefold increase of abundance on the axillary regions and higher diversity on DD submammary areas. An increase of *C. simulans* abundance is characteristic on DD skin. **F** Relative abundances of DD representative taxa proportionally increasing or **G** decreasing with odour intensity. The statistical significance was calculated using Kruskal–Wallis and Wilcoxon-Mann–Whitney tests, respectively, for multiple group and pairwise comparisons. The asterisks indicate statistically significant differences and correspond to *p ≤ 0.05, **p ≤ 0.01, ***p ≤ 0.001, and ****p ≤ 0.0001

DD malodour exerts a strong impact on patients’ lives leading to social isolation in many cases [5]. Taking into consideration that body odours are mainly attributed to bacterial metabolites [23], we sought to investigate the consequences of a DD microbiome dysbiosis on the associated malodour. Correlation analysis of the odour score with other DD characteristics revealed a significant positive correlation between odour intensity and DD severity scores and a negative association with α-diversity values (Fig. 3A). This indicates that in the severe status of DD, where microbial diversity is strongly affected, patients are most susceptible to suffer from body malodour.

We next evaluated the contribution of identified key microbial taxa to DD malodour. Correlation analysis revealed strong positive correlations particularly for *S. aureus*, *S. warneri* and *S. dysgalactiae*, whereas weaker associations were detected for *C. simulans*, *C. coyleae* and *C. jeikeium*. In contrast, *S. hominis 1333*, *S. epidermidis 2429* and *C. acnes* were negatively correlated with the odour score (Fig. 3B). Since *Corynebacteria* are regarded as the primary source of body malodour [28], we next looked at their distribution on healthy and DD predilection skin locations (AX, SM, IN). Overall, *Corynebacteria* were more abundant in DD compared to healthy skin samples and also displayed higher species diversity, especially in AX and SM regions. In AX skin samples, we observed a more than a threefold increase of *Corynebacteria* mean relative abundance both for NIDS (9.08 ± 10%, *p* = 0.014) and IDS (10.74 ± 11%, *p* = 0.07) compared to healthy skin (3.15 ± 4.6%). Of note and in contrast to the Staphylococci group, the Corynebacteria group counts more members both in NIDS and in IDS compared to healthy AX skin (Fig. 3C). Particularly in SM skin, this group shows more taxa in NIDS and IDS compared to healthy SM samples (Fig. 3C–E). To better understand how single members of the *Corynebacteria* group impact malodour in DD, we analysed their distribution in more detail. *C. simulans* was increased in all affected DD skin areas (IDS), particularly SM-IDS skin compared to the SM control group, while *C. coyleae* was more abundant in control and NIDS submammary samples compared to SM-IDS. Also *C. tuberculostearicum* showed a noticeable increase of relative abundance in axillar NIDS (*p* = 0.005) and IDS (*p* = 0.07) skin compared to controls (Fig. 3C–E; Fig S5, D). Of note, the DD patient with the highest objective severity score (ODD = 80) also displayed the highest odour score of 10 and was colonized by *C. simulans* at a mean relative abundance of 27% and 28% on AX and SM, respectively. Another patient with an elevated ODD score (ODD = 50.5) and the second highest odour intensity score of 9 also showed a high *C. simulans* relative abundance of 32% on SM skin.

Study participants with DD were further divided into "mild " (*n* = 5), "moderate " (*n* = 4) and "intense " (*n* = 4) odour groups based on their odour score and analysed with regard to the distribution of key taxa based on this parameter. In line with the obtained correlation data, *C. simulans*,* S. dysgalactiae*,* S. aureus* and *S. warneri* were dominant in the “intense” odour group, while key commensals, particularly *S. hominis* and *C. acnes*, were completely depleted from microbiomes of patients with high odour scores (Fig. 3F, G). Other *Corynebacterium* species such as *C. bouchesdurhonense* and *C. appendicis* were more frequent in the mild odour group, whereas *C. coyleae*, *C. jeikeium* and *C. massilense* were abundant in the moderate odour group. *C. tuberculostearicum* was slightly increased in both mild and moderate odour groups (Fig. S7). In light of these data, DD skin displays a dysbiotic shift with abundant species from the genera *Corynebacterium*, *Staphylococcus* and *Streptococcus* that strongly correlate with the malodour intensity.

### DD skin enrichment in epidermal repair and host defence pathways in response to skin dysbiosis

**Fig. 4:**
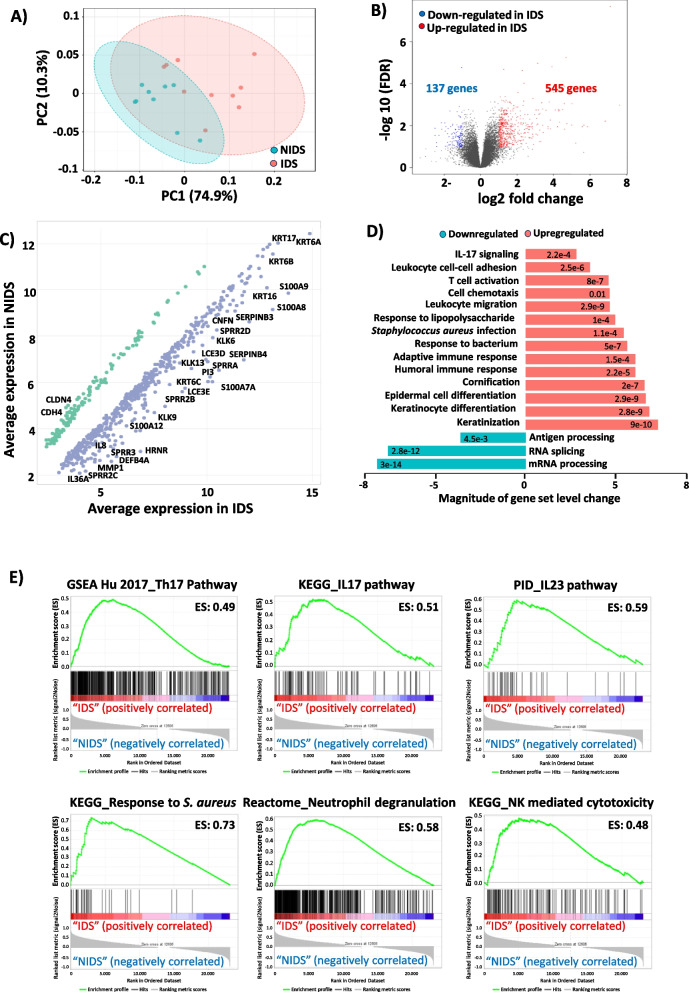
Epidermal repair and Th17 inflammatory pathways are upregulated in response to DD dysbiosis. A Principal component analysis (PCA) shows an altered gene expression profile of inflamed (IDS) compared to non-inflamed (NIDS) DD skin. **B** Volcano plot of fold expression change (FCH) vs false discovery rate (FDR) of the 2000 most varying genes in IDS versus NIDS transcriptomes reveals a significant upregulation of 545 differentially expressed genes (DEGs) and downregulation of 137. This plot displays the significance versus fold-change on *Y* and *X* axes, respectively. **C** Scatter plot of IDS versus NIDS gene expression profiles highlighting representative regulated transcripts. **D** Gene Ontology (GO) analysis of DD skin shows an enrichment of epidermal repair pathways and immune responses to pathogens with strong Th17 signatures. **E** Gene set enrichment analysis (GSEA) plots displaying representative DD-enriched immune pathways. To the far left (red) the plot shows a correlation of the gene set with the IDS phenotype and to the far right a correlation with the NIDS. The vertical black lines indicate the position of each gene within the ranked gene list. Every time a gene from the gene set is detected a hit is plotted. The green curve represents the running sum of the enrichment score of the GSEA. ES (enrichment score), KEGG (Kyoto Encyclopedia of Genes and Genomes) and PID (pathway interaction database)

To investigate the possible consequences of the peculiar microbiome composition in DD and to better explore the host-microbiota interplay leading to skin inflammation, we next investigated the regulation of gene transcription in DD skin. To this end, we performed a transcriptomic profiling of skin biopsies taken from ten participants (9 NIDS and 10 IDS). The principal component analysis (PCA) revealed a distinct clustering of the NIDS and IDS transcriptomes (Fig. 4A) with 545 significantly upregulated and 137 significantly downregulated genes in IDS versus NIDS transcriptomes as depicted in the volcano plot performed on the 2000 most varying genes (Fig. 4B). Scatter plot of IDS versus NIDS gene expression profiles revealed that most of the upregulated transcripts belong to the innate inflammatory response and keratinization processes (Fig. 4C). Gene Ontology (GO) analysis showed an upregulation of epidermal repair pathways with increased expression levels of genes associated with keratinocyte differentiation and cornification. Also, genes associated with defence mechanisms against bacteria especially *S. aureus* were upregulated, including immune genes and a prominent *IL17* signature. In contrast, pathways linked to antigen processing or RNA splicing and processing were downregulated (Fig. 4D). To further explore DD pathogenesis, we next performed a gene set enrichment analysis (GSEA). GSEA data revealed an enrichment of IDS transcriptome in genes related to antimicrobial defence mechanisms with a strong Th17 signature and its associated *IL17* and *IL23* pathways. Furthermore, neutrophil degranulation and NK-mediated cytotoxicity signatures were increased in IDS skin. The mounted immune response seems to be mainly directed against the skin colonization by *S. aureus* and associated dysbiosis as depicted in the enrichment plot of this pathogen (Fig. 4E). Additionally, characteristic DD pathways of keratinocytes hyperproliferation, cornification and apoptosis were upregulated, whereas tight junction proteins displayed a dysregulation (Fig. S8A). Comparison of DD transcriptome with other frequent skin disorders revealed a particular enrichment in psoriasis and atopic dermatitis signatures, respectively, sharing Th17 and response to *S. aureus*-infection pathways (Fig. S8B).

**Fig. 5:**
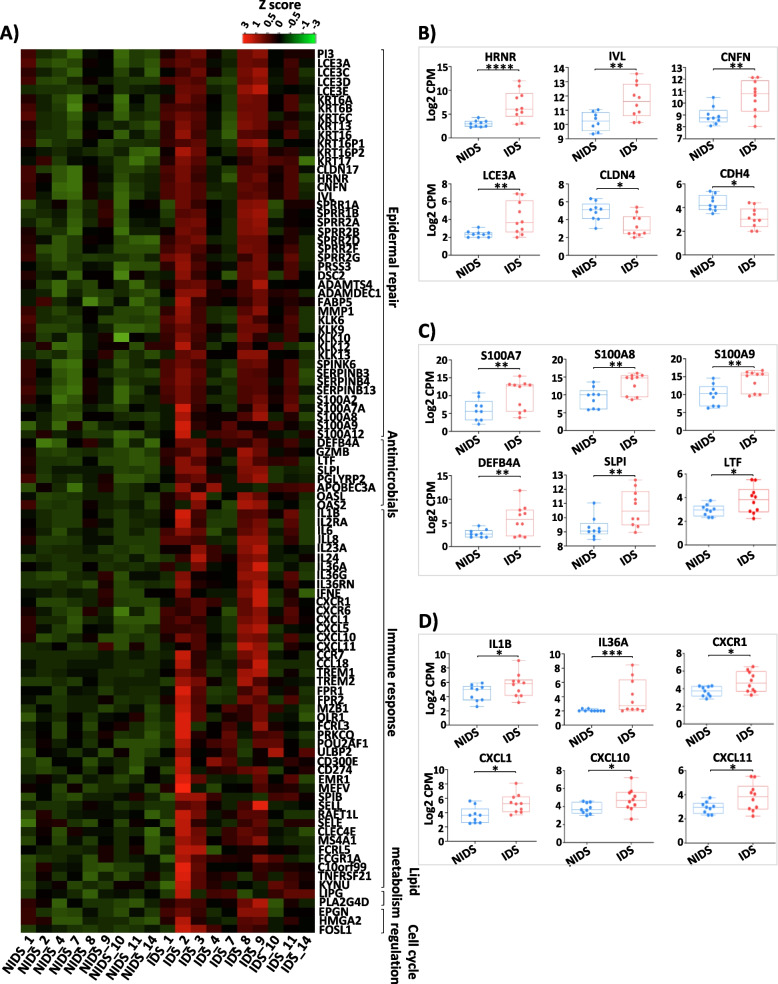
Cutaneous transcriptome reveals characteristic DD signatures. **A** Heat map plot of top 100 genes with high expression levels in DD skin lesions. **B** Normalized count per million (CPM) of selected gene representatives of the epidermal repair cluster **C** antimicrobial defence and **D** immune response clusters. DESeq2 package was used to identify the DEGs using a cutoff threshold of FDR < 0.05, adj. *p* < 0.05 and fold change (FCH) > 1.5. The asterisks indicate statistically significant differences and correspond to *p ≤ 0.05, **p ≤ 0.01, ***p ≤ 0.001, and ****p ≤ 0.0001

In line with these observations, differentially expressed genes (DEGs) between NIDS and IDS (Fig. 5A: top 100 genes) showed a significant upregulation of genes (false discovery rate (FDR) < 0.05, adj. *p* < 0.05 and fold change (FCH) ≥ 1.5) involved in keratinocyte differentiation and cornification pathways (*PI3*, *SPINK6*, *KLK6*, *KLK9*, *KLK12*, *KLK13*, *KRT6A*, *6B*, *6C*, *KRT13*, *KRT16*, *KRT17*, *IVL*, *LCE3A*, *LCE3C*, *LCE3D*, *LCE3E*, *HRNR*, *CNFN*, *MMP1*, *SPRR2A*, *2B*, *2F* and *PRSS3*), antimicrobial defence (*S100A7*, *S100A8*, *S100A9*, *S100A12*, *DEFB4A*, *SLPI*, *LTF*) and chemokines (*CXCR1*, *CXCR6*, *CXCL1*, *CXCL5*, *CXCL10*, *CXCL11*, *CCL18*) as well as immune cytokines and associated receptors (*IL1B*, *IL36A*, *IL36G*, *IL36RN*, *IL24*, *IL6*, *IL8*, *IL23A*, *IL2RA*). Other inflammation-associated genes such as triggering receptors expressed on myeloid cells (*TREM*)1 and *TREM2*, mediating infection-related inflammation [29], genes linked to antiviral response (*APOBEC3A*, *OASL*) and genes not involved in counter-infection mechanisms but associated with cell cycle regulation (*HMGA2*, *CCNB1*, *FOSL1*) and lipid metabolism (*LIPG*, *PCSK9*, *APOL1*, *FABP5*) were also upregulated (Fig. 5A). To better depict expression levels in individual samples and the variance of expression within the groups, normalized counts per million (CPM) are displayed for representative genes. These genes are involved in keratinocyte differentiation and repair (Fig. 5B), antimicrobial defence (Fig. 5C) and immune response pathways (Fig. 5D). Of note, barrier genes including claudin-4 (*CLDN4*) and cadherin-4 (*CDH4*) were noticeably downregulated in DD skin. The *IL17* pathway seems to play an important role in the pathogenesis of DD skin inflammation and thus possibly in DD exacerbations. In fact, many of the key upregulated genes in IDS skin, particularly those involved in immune responses to pathogens, are highlighted on the KEGG *IL17* pathway (Fig. S9). To confirm that immune response genes are also transcribed into protein and to investigate if these involve infiltration of T cells into DD skin, we performed immunohistochemical examinations of collected biopsies for CD4 and IL-17A expression. Indeed, we detected cellular infiltration by CD4 T cells and by IL-17A^+^ cells, particularly in severe DD cases (Fig. S10). Taken together, gene expression driving epithelial repair and anti-microbial responses is upregulated in inflamed DD skin with a central role of the *IL17* pathway.

### DD skin transcriptomics reveals specific gene regulations in response to dysbiotic microbiota

**Fig. 6:**
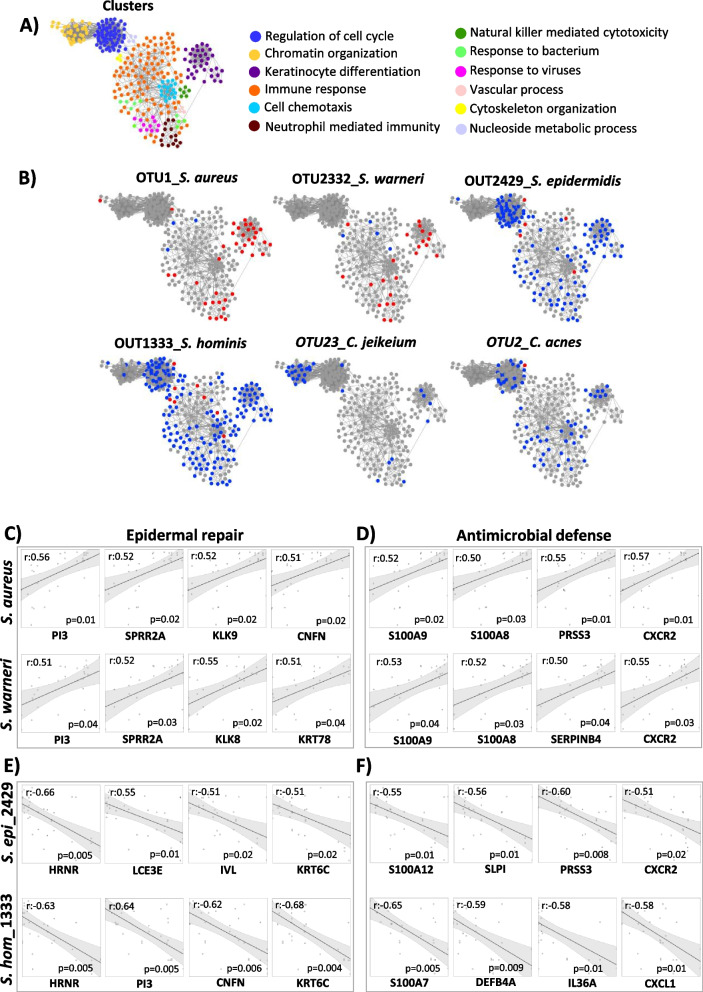
Pathogens and commensals signatures in Darier skin. **A** Network analysis of DD representative gene clusters. The gene network of differentially expressed DEGs between IDS and NIDS was designed on Cytoscape 3 and gene clusters were identified with ClusterViz using the molecular complex detection algorithm (MCODE). **B** Representative gene clusters with taxa-associated host signatures. Key bacterial species on DD skin show significantly positive (red) or negative correlations (blue) with genes from different functional clusters. Linear correlations between epidermal repair or antimicrobial defence clusters reveal significant correlations with **C**, **D** key pathogens and **E**, **F** skin commensals relative abundances. Correlations were calculated on R program and significance was adjusted for FDR

With the delineation of microbial compositions and gene regulations that argue for a dysbiosis-driven inflammation underlying DD exacerbations, an association for single taxa and their respective inflammatory response was investigated. To this end, we constructed a network including the most differentially expressed genes between IDS and NIDS areas distributed over 12 main clusters (Fig. 6A, Fig. S11). Only the most abundant taxa and their associated host signatures are displayed (6 taxa) (Fig. 6B). *S. aureus* and *S. warneri* showed significant positive correlations with a number of host genes, particularly involved in keratinocyte differentiation (*PI3, SPRR2A, KLK8, KLK9, CNFN* and *KRT78*) or antimicrobial defence (*S100A8, S100A9, PRSS3, SERPINB4* and *CXCR2*), in addition to neutrophil associated immune genes (Fig. 6C, D). In contrast, skin commensals particularly *C. acnes*, *S. epidermidis* and *S. hominis* were rather associated with a downregulation of genes belonging to the epidermal repair cluster (*HRNR*, *PI3*, *CNFN*, *IVL* and *KRT6C*) (Fig. 6E). Moreover, the innate immune response clusters (*S100A7A*, *S100A12*, *DEFB4A*, *SLPI*, *PRSS3*, *IL36A*, *CXCR2* and *CXCL1*) (Fig. 6F) were less induced in DD skin colonized by these putatively beneficial microbes. Also the observed negative correlation of cell cycle-related genes in response to these commensals points toward an ability to reduce the epidermal turnover via limitation of the inflammation-induced skin damages. *C. jeikeium* exhibited a different pattern of negative correlation with genes involved in chromatin organization (Fig. 6B). The obtained data indicate that taxa present in the DD microbiome induce specific signatures in the skin transcriptome. Among them, an upregulation of the epidermal repair pathways was strongly induced in response to *S. aureus* and *S. warneri*, probably as an attempt to control DD inflammation and associated skin damages. These bacteria also displayed strong positive correlations with genes of neutrophil-mediated immunity cluster. In contrast, commensal taxa particularly *S. epidermidis* and *S. hominis* seem to exert protective actions against the DD-induced skin damages.

## Discussion

Chronic inflammatory skin diseases are mostly based on a complex genetic inheritance and the development of skin inflammation is a consequence of the interaction of often multiple alterations. Analyses of skin inflammation in DD, which is based on a single mutated gene primarily affecting the skin barrier, in contrast, allow much more stringent conclusions. Indeed, analysis of β-diversity revealed significant changes of the microbiome in DD compared to healthy skin. Importantly and unlike atopic dermatitis or psoriasis [30, 31], microbiota in NIDS and IDS were highly similar. Thus, the loss of *ATP2A2* function determines changes in the composition of the cutaneous microbiota, that are, at least at the predilection sites, specific for DD. Nevertheless, only IDS was characterized by a significant drop of α-diversity, reflecting the strong colonization by *S. aureus*, similar to findings from atopic dermatitis cohorts [32]. In addition, our data revealed a significant increase of *S. warneri* abundance on DD lesions, thriving together with *S. aureus* in a possibly synergetic manner. Although commonly labelled as a commensal and reported as prevalent in dental and nasal cavities [33], *S. warneri* was shown to cause opportunistic infections [34]. Both *Staphylococcus* species were associated with DD inflammation and thus appear as microbial markers of DD exacerbations with skin inflammation. Furthermore, we detected a tenfold decline of *C. acnes* relative abundance in DD skin samples, a finding similar to the well-described drop of this species in patients with atopic dermatitis [30] and psoriasis [35]. Noteworthy, the DD phenotype usually develops during puberty, even though the genetic mutation is present from birth [5]. Lipophilic Cutibacteria are known to colonize sebaceous skin areas during this period of life. Benefitting from sebaceous secretions, they remain at stable proportions through adulthood [11]. The drop of *C. acnes* abundance on DD-affected skin argues in favour of a dysbalanced microbiome already in puberty with impaired skin colonization by this bacterium, allowing an expansion of Staphylococci. Indeed, propionic acid production by *C. acnes* was reported to decrease skin pH limiting the growth of most cutaneous pathogens including *S. aureus* [36]. *S. epidermidis* abundance was only slightly affected, in contrast to atopic dermatitis where this bacterium mostly parallels the *S. aureus* expansion [37]. Both *C. acnes* and *S. hominis* showed positive correlations with α-diversity and were inversely correlated with DD severity scores. In addition, they displayed negative correlations with *S. aureus* and *S. warneri*, indicating inhibitory potential towards these *Staphylococci*, in line with several reports unveiling their beneficial effects [38, 39]. The dysbiosis we observed on DD skin was also accompanied by a loss of potentially beneficial commensals such as *Micrococcus luteus*, *Moraxella osloensis* and *Paracoccus yeei*, corroborating observations from previous atopic dermatitis cohorts [32, 40].

The human body malodour is a consequence of the microbial biotransformation of odourless secretions into volatile odorous compounds [28]. In order to investigate the role a dysbalanced microbiome could play in DD-associated malodour, we explored the distribution of key taxa according to odour intensity in our DD cohort. Obtained data revealed that odour intensity positively correlates with DD severity scores and negatively with the microbial diversity, pointing towards a role of a dysbiotic microbiome in malodour. Interestingly Corynebacteria, often linked to axillary malodour in healthy individuals [41], displayed a threefold increase on axillaries of DD patients, in contrast to atopic dermatitis skin, where this group is often drastically reduced [42]. Our observation corroborates findings from a large DD cohort (163 patients), reporting malodorous papillomatous masses on patients’ axillae and groins [5]. Analysis at the species level revealed increased proportions of *C. simulans* on the submammary and axillary areas of DD patients with the highest malodour scores, showing the importance of this bacterium in malodour generation. Volatile fatty acids (VFAs) and thioalcohols have been reported as the main cause of axillary malodour [28]. 3-Methyl-2-hexenoic acid (3M2H), being the dominant VFA in axillar perspiration is released from the skin by corynebacterial Na-acylglutamine-aminoacylase, while 3-methyl-3-mercaptohexan-1-ol thioalcohol is produced by the action of their carbon–sulphur (C–S) β-lyases. Both metabolites are the primary causal molecules of underarm odour [43, 44]. It has been reported in addition, that *Staphylococcus* species can catabolize branched aliphatic amino acids to generate odorous metabolites, such as isovaleric acid, associated with axillary malodour [45]. Albeit Corynebacteria can metabolize skin fatty acids at a higher rate than *Staphylococci* [28], we detected much higher abundances of the latter group on DD lesions. This finding is supported by previous studies demonstrating that members from both genera strongly correlate with odour intensity [46]. Our data also showed a significant correlation between *S. dysgalactiae* and DD malodour. In line with that, infections of intertriginous skin folds by β-hemolytic *Streptococci* have been associated with a distinctive foul odour for children suffering from streptococcal intertrigo [47]. In contrast, beneficial skin residents such as *S. hominis*, *S. epidermidis* and *C. acnes* displayed negative correlations with DD malodour, corroborating previous studies on axillary malodour in healthy individuals [23, 48]. The inhabited skin site plays an important role in offering necessary substrates for odour-generating bacteria. Indeed, areas such as axilla and groin are characterized by higher pH values in addition to increased moisture and nutrient supply favouring the bacterial overgrowth [49, 50]. This observation is supported by studies on other diseases characterized by high microbial loads on the intertriginous areas and associated with malodour such as streptococcal intertrigo [47] and hidradenitis suppurativa [51]. In light of these data, the dysbiotic shift observed in DD skin, with an overgrowth of species from Corynebacteria, Staphylococci and Streptococci genera, most likely plays a major role in DD associated malodour.

To investigate how the observed microbial dysbiosis on DD skin is linked to the development of skin inflammation and disease exacerbation, we next performed a transcriptomic profiling of NIDS and IDS skin samples and looked for gene signatures involved in the host-microbiota interplay. DD skin damages seem to be compensated by an accelerated keratinocytes turnover with the upregulation of genes associated with keratinization and cornification processes. Besides their important role in the keratinization process, some of these proteins display defensin-like properties against a broad spectrum of bacteria, including *S. aureus*, as reported for *LCE3* [52], *KRT6A* [53] and hornerin [54]. Furthermore, inflamed DD skin displayed an increase of proteases, especially matrix metallopeptidase 1 (*MMP1*), serine protease 3 (*PRSS3*) and kallikrein serine proteases *KLK6*,* 9*,* 12*,* 13* that are able to cause barrier damages when overexpressed and which actions are mainly neutralized by *SPINK6* [55] and serpins. The upregulated S100 proteins (*S100A7*,* A8*,* A9* and *A12*) were shown to exhibit strong antimicrobial activities against several pathogens and to act as neutrophil and T cell chemoattractants in addition [56, 57]. Moreover, our data showed increased expressions of conventional neutrophil (*CXCR1*, *CXCL1*, *CXCL5*) and T cell (*CXCL10*, *CXCL11*, *CCL18*) chemoattractants. The accumulation of neutrophils in DD-inflamed skin may add to the elevated expression levels of antimicrobials as defensin beta-4A (*DEFB4A*) and lactotransferrin (*LTF*), which are strongly produced by these cells [58, 59]. Activated neutrophils are able to cause considerable collateral damage by releasing numerous proteases, whose effect is mainly counterbalanced by the peptidase inhibitor-3 (*PI3*), a product of Th17 cells [60] and the secretory leucocyte peptidase inhibitor (*SLPI*) [61]. The expression of both is upregulated in parallel with *S. aureus* and *S. warneri* colonization on DD skin, suggesting that they are triggered to prevent protease-mediated tissue damages due to an excessive innate immune response. Likewise, several pro-inflammatory cytokines were upregulated, particularly the IL-1 family cytokines with *IL1b* playing a central role in neutrophils and lymphocytes activation and with *IL36A* and *IL36G* acting on keratinocytes, dendritic cells and T cells [59]. We also detected significant increases of *IL23A* and *IL24* involved in the IL-23 signalling pathway in addition to *IL6* and *IL8* (*CXCL8*) previously shown to be induced in response to S100A12 [62]. This cytokine milieu explains the dominant Th17 signatures as it has been reported that exposure to IL-1β and IL-23 orchestrates the so-called type 3 immune responses [63]. The assessment of host-bacteria interactions showed a positive correlation between *S. aureus* and *S. warneri* with a number of key genes, particularly belonging to the epidermal repair cluster and genes related to neutrophil and Th17-driven inflammation. The IDS skin with its damaged barrier and, as shown in our study, poor expression of inter-cellular adhesion proteins [64], particularly of *CLDN4* and *CDH4* must facilitate the passage of pathogens associated molecular patterns (PAMPs) produced in high amounts by its microbial burden, thereby exacerbating skin inflammation. This explains the clear differences of NIDS and IDS transcriptomes despite the strong similarities of their microbiome profiles. Of note, a downregulation of *CLDN4* was reported in AD, showing furthermore a negative correlation with *S. aureus* relative abundance [65]. Likewise, a decrease in CDH4 expression was observed upon stimulation of an AD phenotype in NC/NgA mice using house dust mites [66]. Although other key barrier genes such as CLDN1, CDH1, KRT1 and KRT10 were expressed at higher levels in DD skin compared to CLDN4 and CDH4, they did not show however significant differences between NIDS and IDS and therefore were not in the focus of our study. On the other hand, skin commensals such as *S. hominis* and *S. epidermidis* displayed negative correlations with these clusters, suggesting that innate immune ligands of commensal bacterial origin are much less pro-inflammatory than those from *S. aureus* as also shown previously [67]. Previous studies on atopic dermatitis similarly reported a significant upregulation of pathways involving defence mechanisms particularly against *S. aureus*, with increased expression levels of *S100* proteins, *DEFB4A*,* MMPs*,* SLPI* and *LTF*. Nevertheless, the atopic dermatitis characteristic Th2 or type 2 signature (*IL4*, *IL13*,* IL22*,* TSLP*,* CCL13*,* CCL17* and *CCL26*) [68] was absent from DD skin, despite the strong colonization by *S. aureus* observed in both diseases. DD-regulated genes particularly displayed similarities with psoriasis showing a prominent Th17 or type 3 signature and upregulation of DEGs attributable to IL-17 and IL-23 signalling [69].

In light of these data, interventions correcting the dysbiosis in DD via microbiome transplantation or selective promotion of beneficial bacteria’s growth, especially *S. hominis* and *C. acnes*, may allow to interfere with the development of inflammation and body odours associated with DD exacerbations.

This study is innovative, because it is for the first time exploring cutaneous microbiome and gene expression in DD skin patterns. Yet, some limitations should be mentioned. In our correlation analysis, we performed a log transformation that removes the compositional constraints from taxonomic data to linearize the existing relations. However, it cannot be completely excluded that these relations remain nonlinear. This highlights the fact that correlation analysis methods originally developed for absolute values could lead to spurious correlations when dealing with relative compositional data. Therefore, these results should not be interpreted in terms of causality. Also, a number of patients still received a retinoid or low-dose naltrexone-based therapy during the study and the treatment’s impact on the skin microbiome could not be fully excluded. Finally, DD is a rare disease and despite this study being the first and largest of its kind in DD, further validation of these data in a larger cohort even including an interventional arm should be one next step in the investigations on DD.

### Supplementary Information

**Additional file 1.** Supplementary Figures.

**Additional file 2.** Supplementary methods.

**Additional file 3:** **Supplementary Table S1.** Cohort general characteristics.

**Additional file 4:** **Supplementary Table S2.** Abundant OTUs on DD skin.

**Additional file 5.** Enriched Pathways in IDS vs. NIDS skin.

**Additional file 6.** Transcriptome raw read counts data.

## Data Availability

The datasets supporting the conclusions of this article are included within the article and its additional files.
