# Correction: Darier’s disease exhibits a unique cutaneous microbial dysbiosis associated with inflammation and body malodour

**DOI:** 10.1186/s40168-023-01665-0

**Published:** 2023-09-14

**Authors:** Yacine Amar, Danielle Rogner, Rafaela L. Silva, Bärbel U. Foesel, Minhaz Ud‑Dean, Ilias Lagkouvardos, Susanne A. Steimle‑Grauer, Sebastian Niedermeier, Susanne Kublik, Manja Jargosch, Matthias Heinig, Jenny Thomas, Stefanie Eyerich, Jakob D. Wikström, Michael Schloter, Kilian Eyerich, Tilo Biedermann, Martin Köberle

**Affiliations:** 1 Department of Dermatology and Allergy, Technical University of Munich, School of Medicine, Munich, Germany; 2 Research Unit Comparative Microbiome Analysis, Helmholtz Zentrum München, Deutsches Forschungszentrum Für Gesundheit Und Umwelt (GmbH), 85764 Neuherberg, Germany; 3 Institute of Computational Biology, Helmholtz Zentrum München, German Research Center for Environmental Health, 85764 Neuherberg, Germany; 4 Department of Informatics, Technical University of Munich, Garching, Germany; 5 Core Facility Microbiome, Technical University of Munich, 85354 Freising, Germany; 6 Center of Allergy & Environment (ZAUM), Technical University of Munich (TUM) and Helmholtz Zentrum München, German Research Center for Environmental Health, Member of the German Center of Lung Research (DZL), Munich, Germany; 7 Dermatology and Venereology Division, Department of Medicine Solna, Karolinska Institutet, Stockholm, Sweden; 8 Dermato‑Venereology Clinic, Karolinska University Hospital, Stockholm, Sweden; 9 Department of Dermatology and Venereology, Medical Center, University of Freiburg, Freiburg, Germany

**Correction** **:** **Microbiome 11, 162 (2023)**

**https://doi.org/10.1186/s40168-023-01587-x**

Following publication of the original article [1], the author reported that the name “Jakob Wikström” should be “Jakob D Wikström”

This has been corrected above and the original article has been updated.
