# Supplementary material for: Darier’s disease exhibits a unique cutaneous microbial dysbiosis associated with inflammation and body malodour
Source: Microbiome. 2023 Jul 26;11:162. doi: 10.1186/s40168-023-01587-x (PMC10369845; doi:10.1186/s40168-023-01587-x)
Supplement: Supplementary file 2 — Additional file 1. Supplementary Figures. [file 40168_2023_1587_MOESM1_ESM.pdf]

A= Severity

Hyperkeratosis / induration of papules

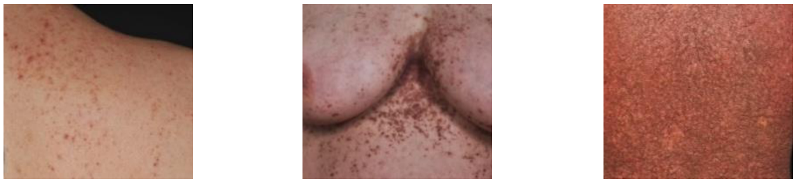

0= none | **1= mild induration of papules** | **2= moderate induration of papules** | **3= severe induration of papules**

Erosions

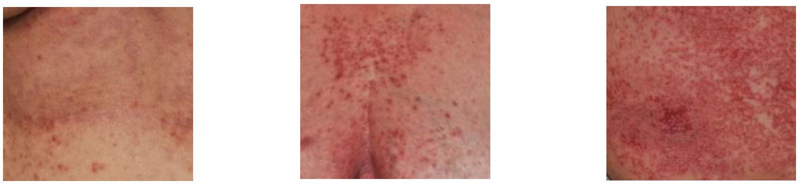

0= none | **1= mild (dry erosions)** | **2= moderate (solitary macerated erosions)** | **3= severe (multiple macerated erosions)**

Severity	Axilla	Inguinal	Submammary	Facial	Other areas
**Hyperkeratosis**	0-3	0-3	0-3	0-3	0-3
**Erosions**	0-3	0-3	0-3	0-3	0-3
**Sum**	A1=	A2=	A3=	A4=	A5=

A= A1+A2+A3+A4+A5

B= Extent

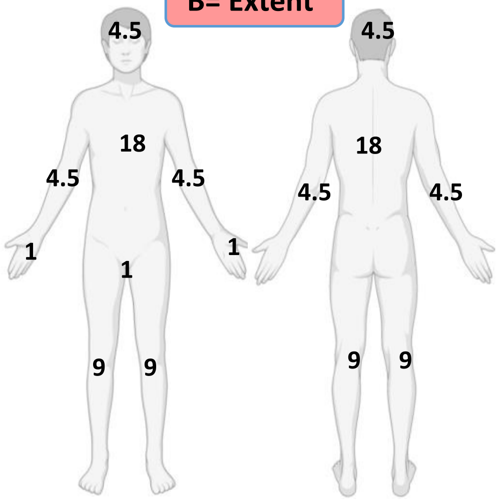

B= Body surface area (%) / 2

C= Subjective

0= no itch	5= distressful itch	10= unbearable itch
0= no pain	5= distressful pain	10= unbearable pain

C= value pain (C1) + value itch (C2)

oDD Score: A/5 + 7B/2

DD Score: A/5 + 7B/2 + C

Supplementary Figure 1: Scoring Darier's disease. A DD score was developed based on the concept of the SCORAD (Scoring Atopic Dermatitis). Consequently, the objective DD (oDD) score combines **A)** severity measures (hyperkeratosis, induration of papules, erosive areas) and **B)** the extent of lesions (total affected skin surface area). **C)** For the global DD score an additional subjective DD score (considering pain and pruritus) is added and sums up to a maximum of 103.

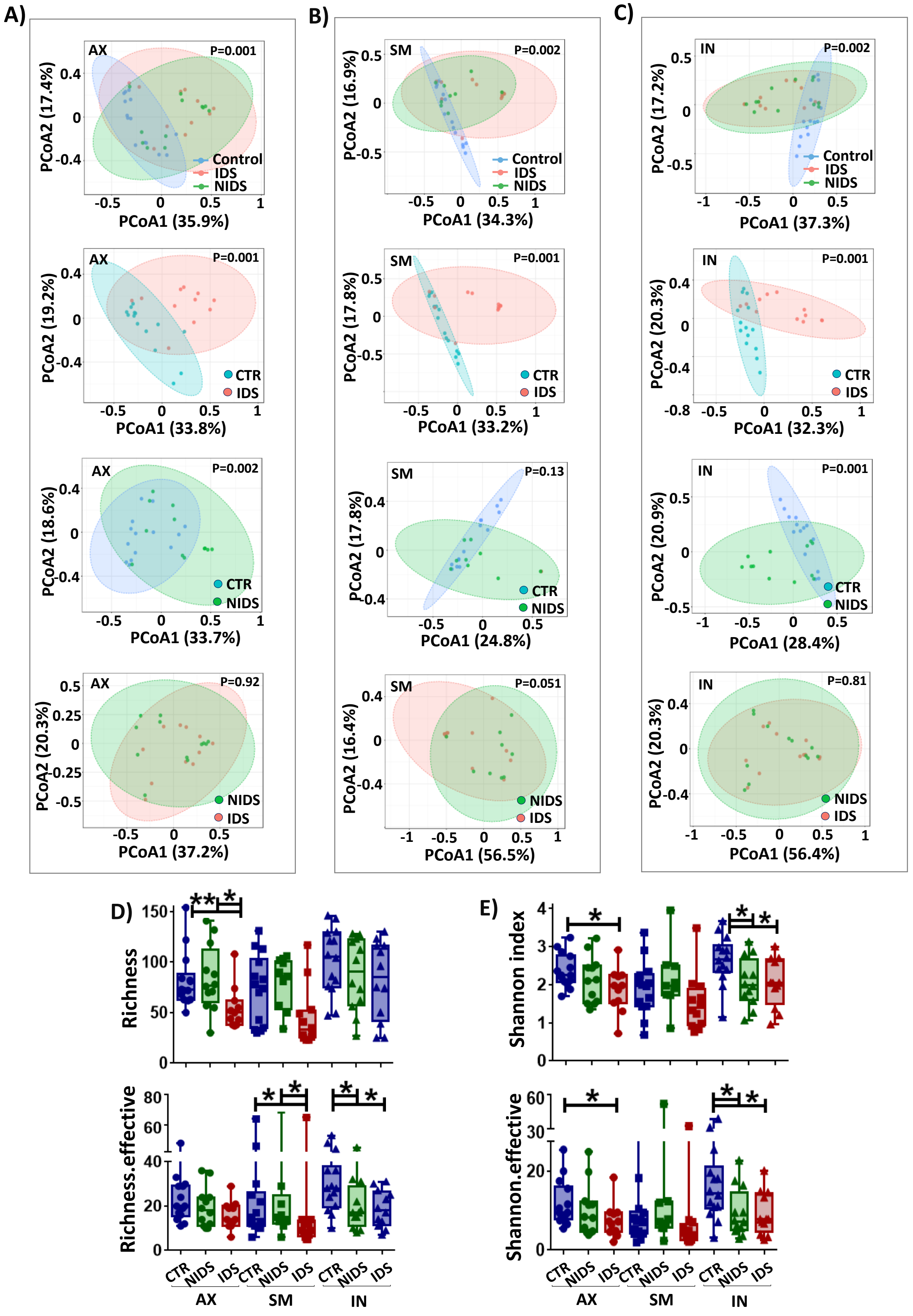

Supplementary Figure 2. Microbial diversity analysis of Darier skin by sampling site. A-C) PCoA (principal coordinate analysis) plots providing a distance based rendering of inter-group taxonomic differences of samples taken from **A)** axillary, **B)** submammary, or **C)** inguinal regions. **D-E**) Intra-group α-diversity was analysed expressed as **D)** richness (number of OTUs), and **E)** Shannon index or their effective values. AX (axillar), SM (submammary), IN (inguinal). CTR (control), IDS (inflamed Darier skin), NIDS (non-inflamed Darier skin). *p≤0.05, **p≤0.01.

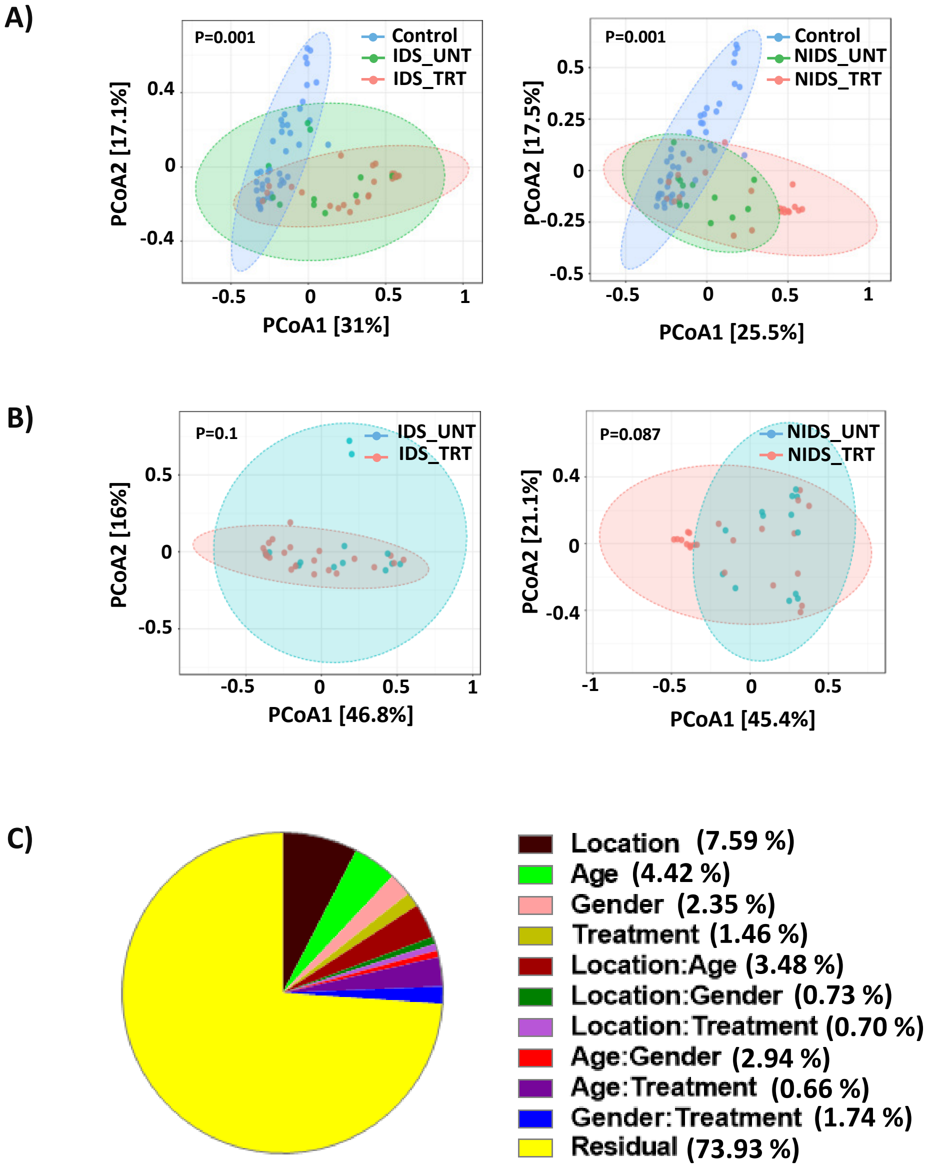

Supplementary Figure 3. Microbiome analysis of Darier skin treated or not with retinoids or low dose naltrexone. A) PCoA (principal coordinate analysis) plot of β-diversity profiles of IDS and NIDS of untreated patients and patients under therapy compared to healthy skin **B)** β-diversity plot displaying treated or untreated IDS and NIDS skin. The Bray Curtis index was used to calculate similarity between samples and PERMANOVA to test the statistical significance based on the distance matrix. **C)** PVCA analysis of the contribution of different covariates to the batch effect. IDS (inflamed Darier skin), NIDS (Uninflamed Darier skin), TRT (treated), UNT (untreated). PVCA (Principal Variance Component Analysis).

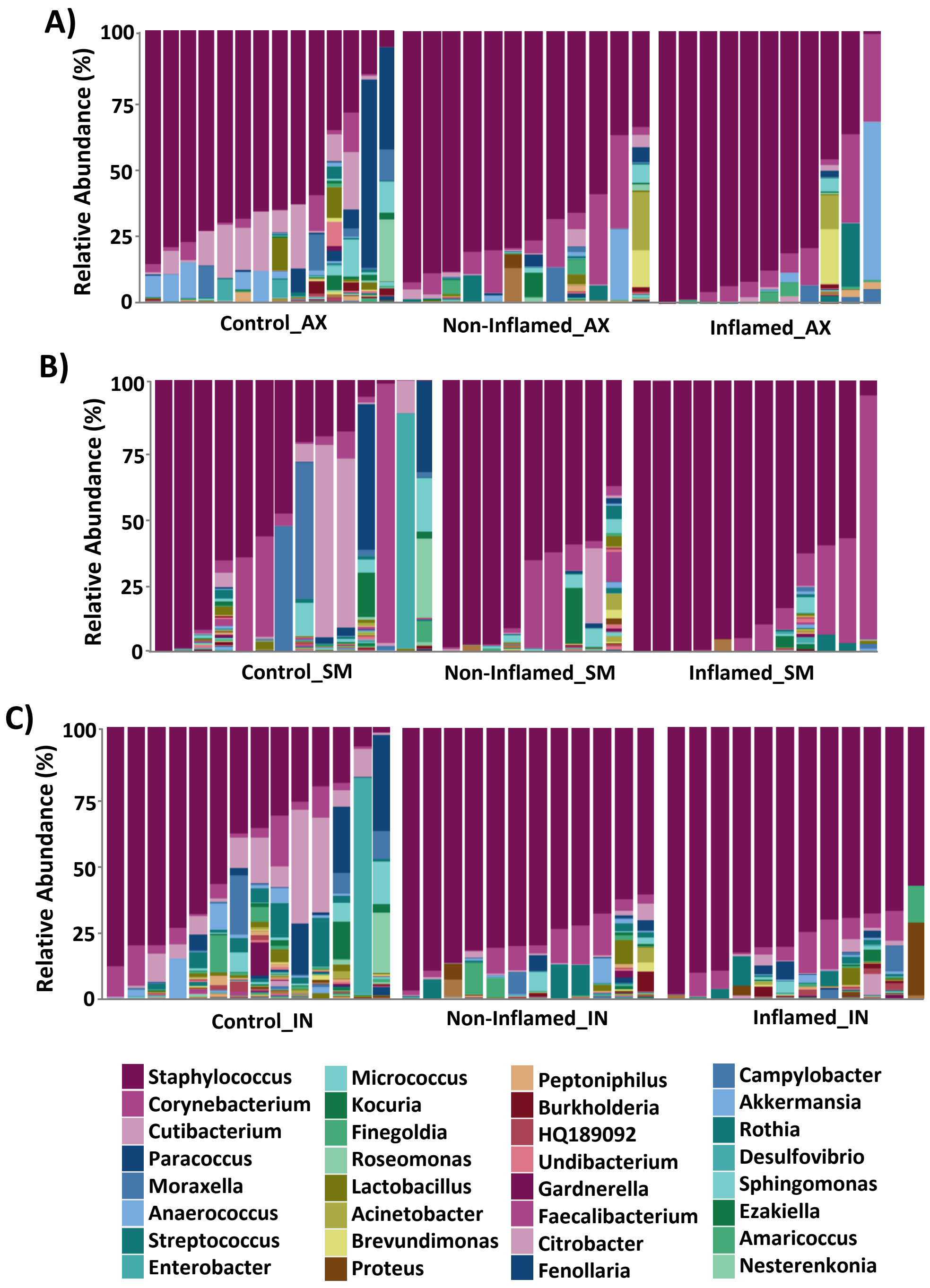

Supplementary Figure 4: Relative abundances of bacterial genera by location. Bar chart of taxonomy binning at genus level of the **A)** axillary, **B)** submammary, and **C)** inguinal areas. The taxonomic composition was assessed by summing up OTUs relative abundances that share the same assignment at a genus level. The Bayesian classifier from RDP database was used for OTUs classification. AX (axillar), SM (submammary), IN (inguinal).

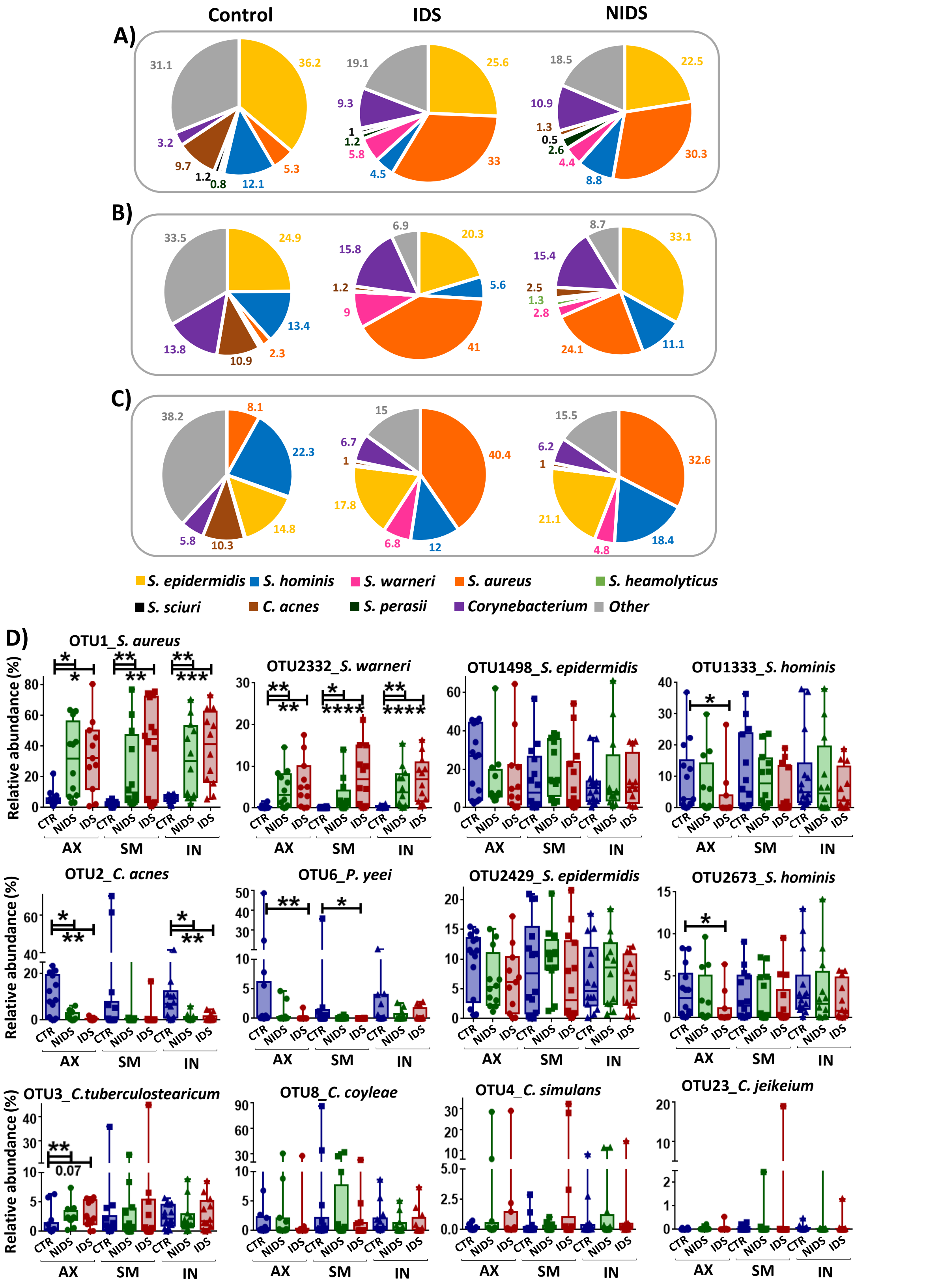

Supplementary Figure 5: Relative Abundances of key bacterial taxa by location. A-C) Pie chart plots showing *Staphylococcus* species, *C. acnes* and *Corynebacterium* group distributions on a **A)** axillary, **B)** submammary, or **C)** inguinal regions. **D)** Relative abundances of OTUs with major shifts, on the different sampled locations. AX (axillar), SM (submammary), IN (inguinal). CTR (control), IDS (inflamed Darier skin), NIDS (non-inflamed Darier skin). *p≤0.05, **p≤0.01, ***p≤0.001.

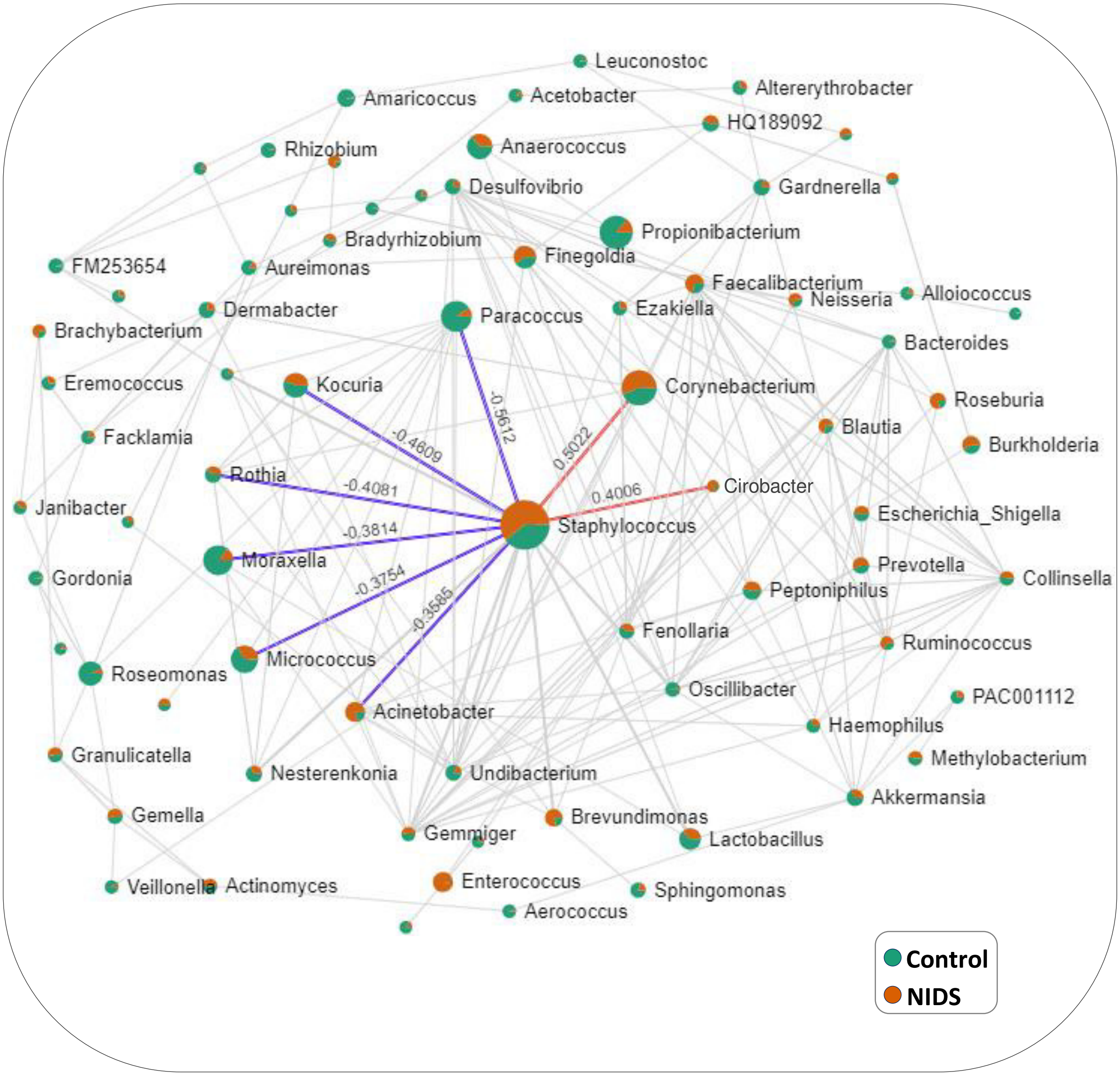

Supplementary Figure 6: Correlation network of microbiome communities in healthy and non-inflamed DD skin (NIDS) at genus level. The SparCC (Sparse Correlations for Compositional data) approach has been used to define network associations. This approach assumes a sparse network, it uses a log ratio transformation and performs iterations to identify taxa pairs that are outliers to background correlations. Each node represents a taxon and its size is proportional to the number of connections. The size of the green fraction of each node indicates the taxon's relative abundance in the control group and the orange fraction in the NIDS, respectively. Taxa are only connected if the correlation meets a p-value cut-off of 0.05 and a correlation coefficient of 0.3. Key correlations with the *Staphylococcus* genus are highlighted in the figure with blue and red lines respectively representing negative and positive correlations.

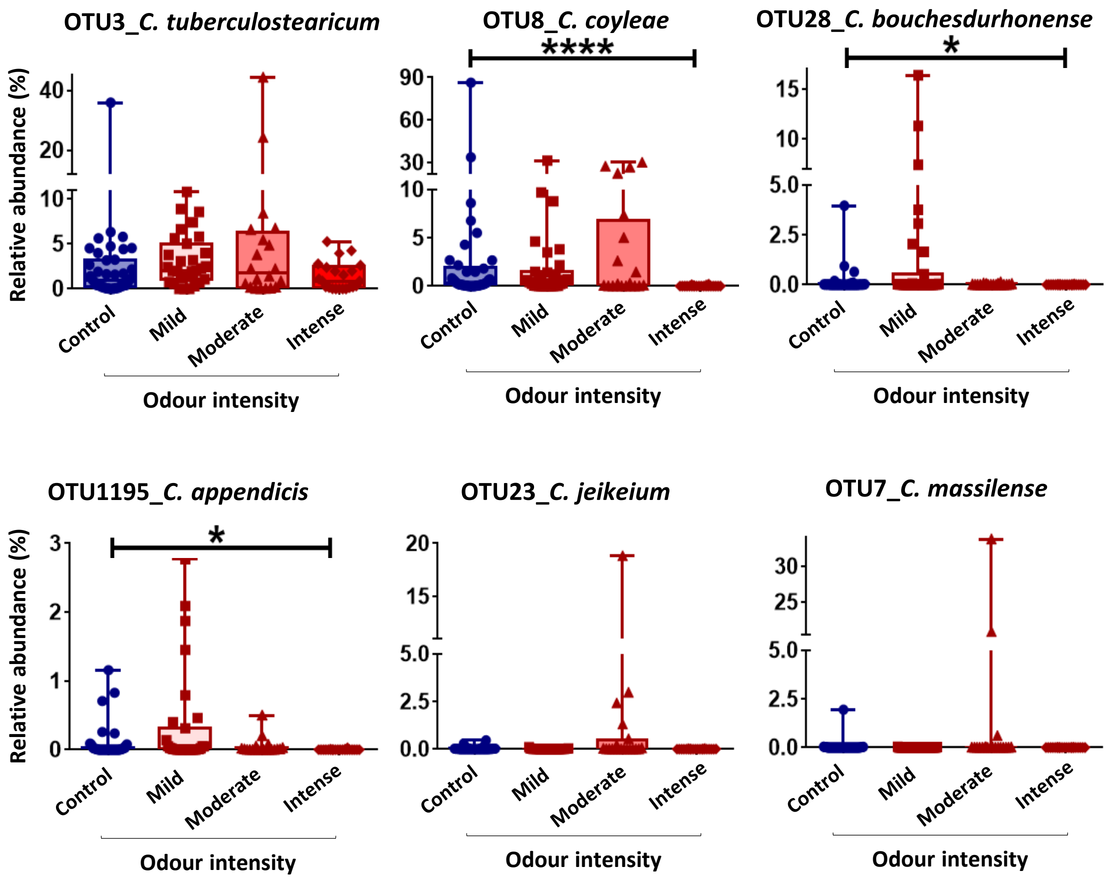

Supplementary Figure 7: Relative abundances of *Corynebacteria* species grouped by DD patients' odour intensity.

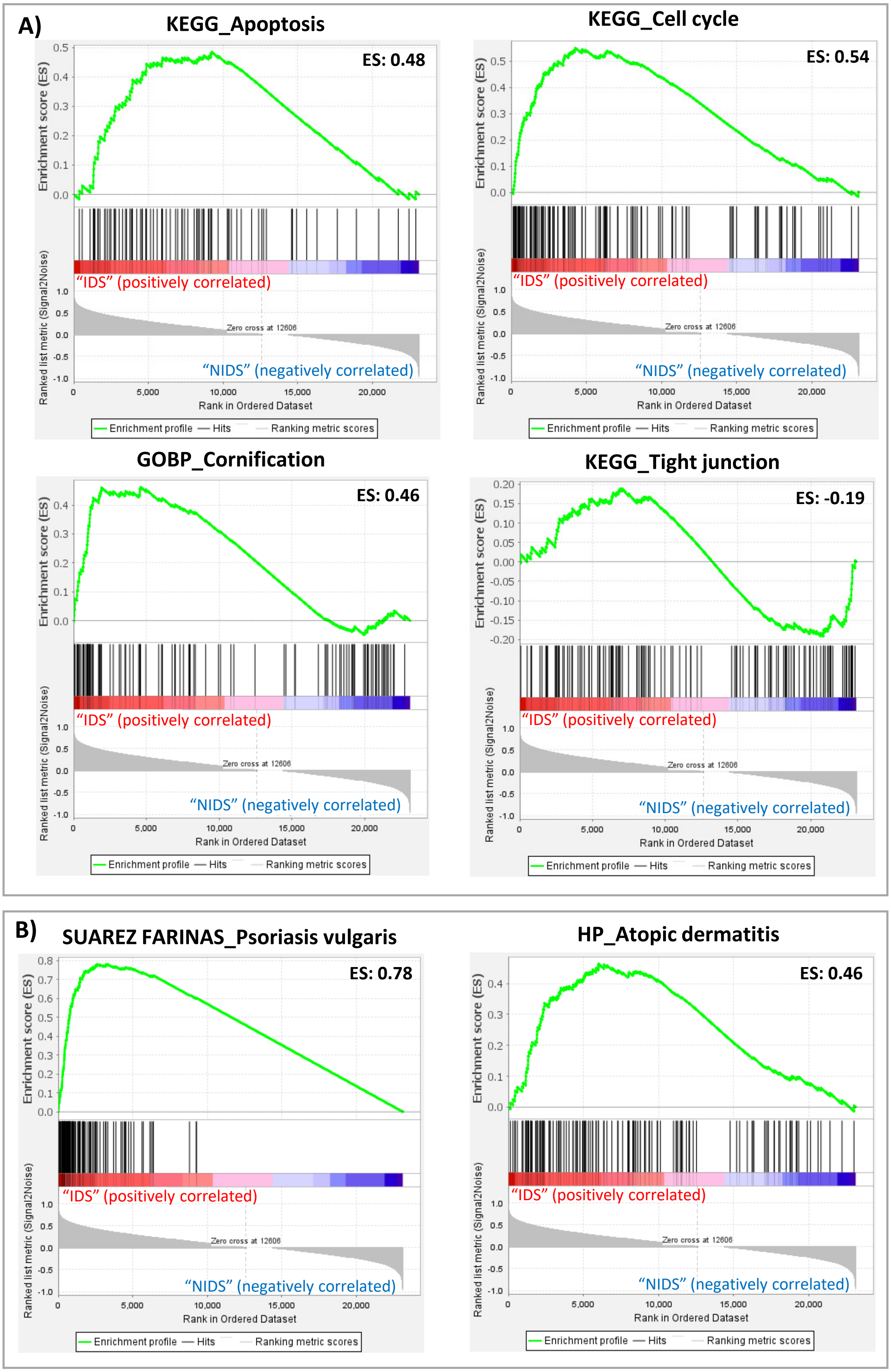

Supplementary Figure 8: Gene set enrichment analysis (GSEA) of DD cutaneous transcriptome. A) GSEA plots of representative enriched DD pathways. **B)** Enrichment plots of psoriasis vulgaris and atopic dermatitis signatures. ES: Enrichment score. IDS (inflamed Darier skin), NIDS (non-inflamed Darier skin). KEGG (Kyoto encyclopaedia of genes and genomes), GOBP (Gene ontology for biological process), SUAREZ FARINAS (Psoriasis cohort), HP (Human phenotype).

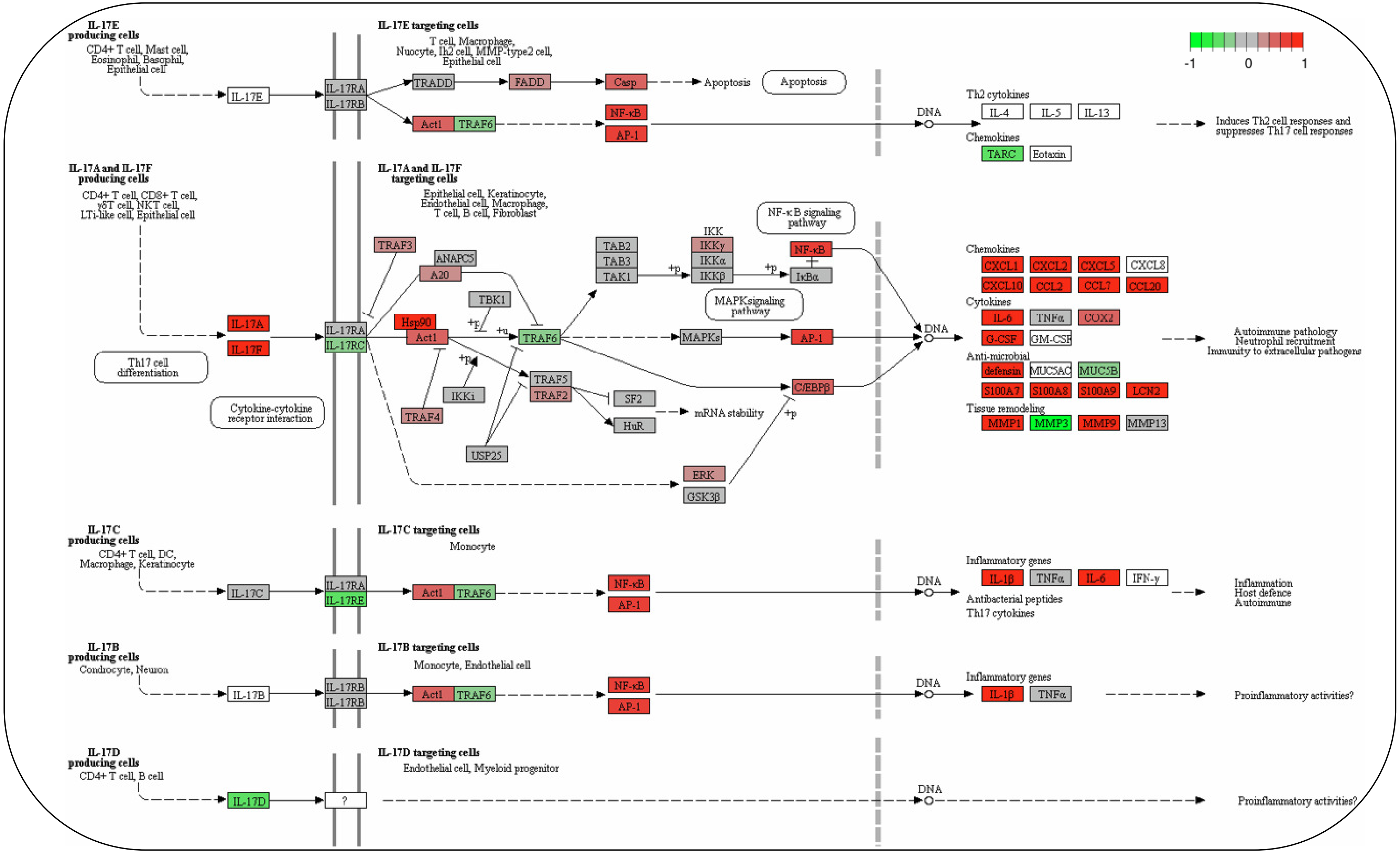

Supplementary Figure 9: KEGG IL-17 signaling pathway highlighting genes upregulated in IDS compared to NIDS DD skin. KEGG (Kyoto encyclopaedia of genes and genomes)

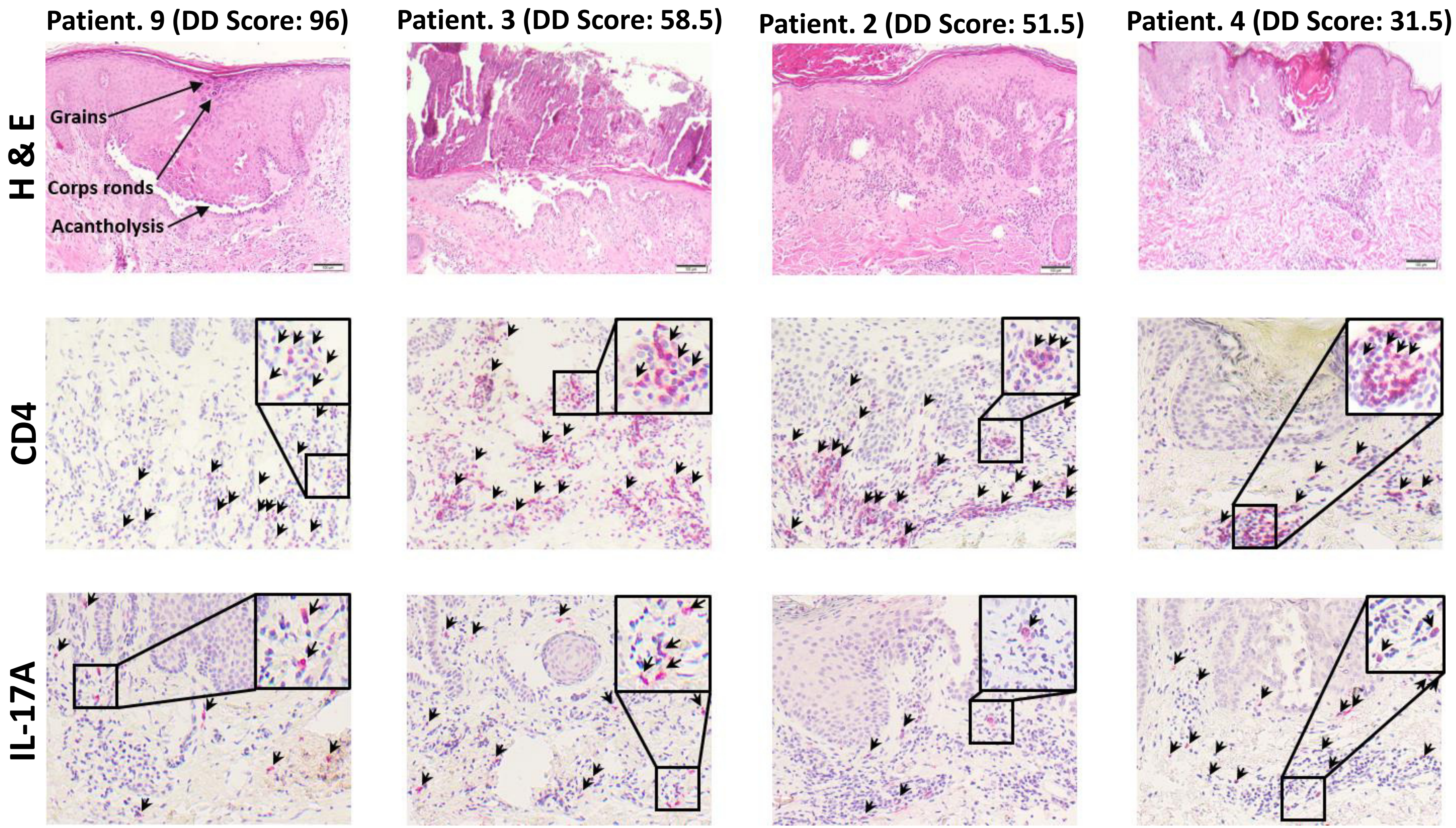

Supplementary Figure 10: Immunohistochemistry of inflamed Darier's skin. Representative histological sections of inflamed DD skin collected from patients with different disease severity scores. Sections were stained with hematoxylin/eosin (top), anti-CD4 (middle) and anti-IL-17A (bottom). The typical DD hallmarks of grains, corps ronds and acantholysis are depicted in the H&E staining (long arrows). Examples of CD4 or IL-17A positive cells or cell clusters are marked by short arrows. Microscopic magnification was set at 10x for H&E and 20x for the other stainings.

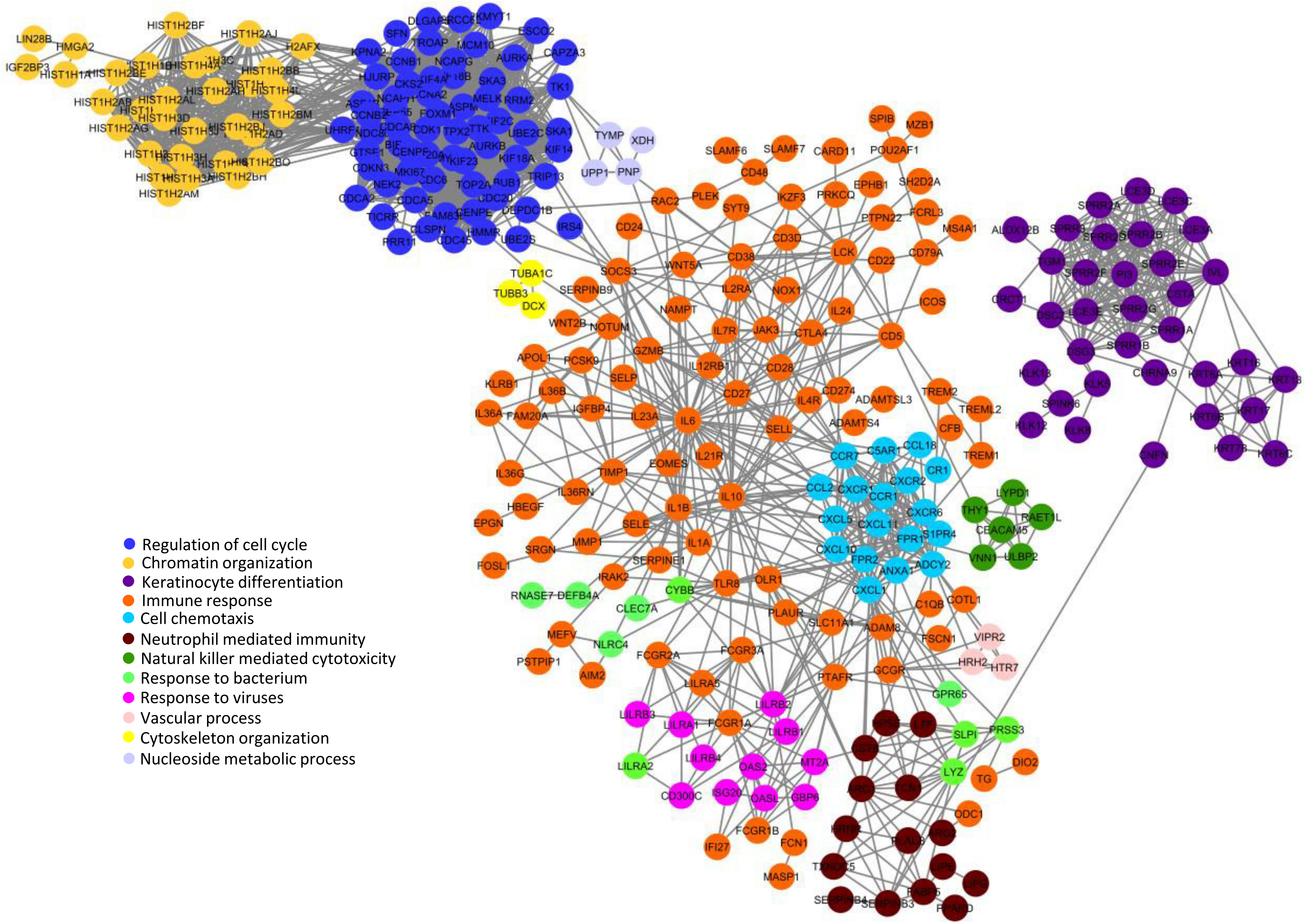

Supplementary Figure 11: Network plot of most differentially expressed genes between IDS and NIDS skin distributed over 12 main clusters. This data plot was generated using Cytoscape 3.
