# Supplementary material for: Darier’s disease exhibits a unique cutaneous microbial dysbiosis associated with inflammation and body malodour
Source: Microbiome. 2023 Jul 26;11:162. doi: 10.1186/s40168-023-01587-x (PMC10369845; doi:10.1186/s40168-023-01587-x)
Supplement: Supplementary file 3 — Additional file 2. Supplementary methods. [file 40168_2023_1587_MOESM2_ESM.pdf]

Supplementary material and methods

Subjects

Study participants were selected according to the inclusion/exclusion criteria approved by the regional government of Oberbayern. An approval by the medical ethical committee and individual informed consent were obtained in advance of the sample collections. Fourteen Darier patients were recruited at the Department of Dermatology and Allergy of the Technical University of Munich and followed up all along the course of this study. Due to a lack of an established scoring approach for DD, a scoring was developed based on a scoring approach for atopic dermatitis, the SCORAD (Scoring of Atopic Dermatitis) (1), and all DD patients were examined accordingly. The objective score for DD (ODD Score) used here includes a position for 'severity' of DD lesions (A: hyperkeratosis, induration of papules, erosions) and a position for 'extent' of DD lesions (B: total affected surface area of affected skin). The global DD score (DD Score) is calculated by summing up ODD and the subjective score (C: pain and pruritus) as detected using a visual analog scale (VAS; Supplementary Figure S1). The PGA evaluation was well aligned with the established ODD scoring and the latter was therefore used for separating study participants into groups of "mild" (ODD-score: <20), "moderate" (ODD-score: 20-35) and "severe" (ODD scores: >35). Furthermore, patients' odour was assessed as this is one of the predominant burdens in DD. The odour intensity was scored by an experienced dermatologist on a scale from zero (not perceptible) to ten (very intense) (2) and DD patients were categorized into odour groups of "mild" (odour score $\leq$3), "moderate" (odour score 4-6) and "intense" (odour score $\geq$7) (Table 1). For the microbial swabs, sample collection guidelines included in the Human Microbiome Project (3) were applied. Participants were asked to not use any topical corticosteroids at least 3 days before sampling and no systemic antibiotics or corticosteroids for at least 4 weeks. They were also instructed to not shower at least 24h to 48 hours before swabs' collection. Inclusion criteria for healthy controls consisted of the absence of any current or prior chronic skin disorders or use of systemic antibiotics in the preceding 6 months.

Specimen collection

Skin swabs were collected from non-inflamed (NIDS) and inflamed (IDS) axillar (AX), submammary (SM), and inguinal (IN) regions of DD patients and corresponding sites of their healthy matched controls. The NIDS swabs were collected from the unaffected skin areas at a distance of 5 to 10 centimetres from the IDS. Samples were harvested using the forensic 4N6FLOQ swabs (COPAN flock technologies, Italy) previously soaked in sterile 0.15M NaCl supplemented with 0.1% Tween-20. Briefly, 6 cm^2 skin areas were swabbed back and forth approximately 50 times applying a firm pressure, then swirled in a 2 ml collection tube containing 400µl sterile 0.15M NaCl solution. Control swabs were

maintained in the air for 20 seconds, then similarly processed to exclude swab's contaminating reads. To minimize sample cross-contamination, a fresh pair of sterile gloves was worn at every sampling, the collected samples are stored at +4°C and processed for DNA extraction within 24hours (4). Microbial DNA was extracted using a benzonase pre-digest approach that we optimized to better estimate the living skin microbiota (5) using the QIAamp DNA Microbiome kit (Qiagen, Hilden, Germany). This approach ensures an efficient elimination of DNA from dead bacteria as well as skin host DNA, thus enabling a better overview of living and active fraction of the skin microbiome. It also uses an optimized combination of mechanical and chemical lysis ensuring effective disruption of both Gram negative and Gram positive bacteria. Bacterial DNA was purified through adsorption to silica membrane columns having undergone prior DNA decontamination processes. DNA samples were collected in 50µl elution buffer and stored at -30°C until further processing. Skin biopsies were collected by the recruiting dermatologist from 9 NIDS and 10 IDS skin areas on ten patients and stored in RNAlater (Sigma-Aldrich, Germany) at -80°C. Total RNA was isolated from bulk biopsies using the miRNeasy Mini Kit (Qiagen, Germany) according to manufacturer's protocol. This kit includes QIAzol Lysis Reagent which combined phenol and guanidine thiocyanate for effective tissue lysis. It also inhibits RNases and remove most of cellular DNA. Purified RNA samples were stored at -80°C until further use.

16S rRNA gene amplification

The following 16S rRNA gene universal primers targeting the V3-V4 regions were used in this study: Forward:S-D-Bact-0341-b-S-17(5'→3')TCGTCGGCAGCGTCAGATGTGTATAAGAGACAGCCTACGGGNGGCWGCAG. Reverse:S-D-Bact-0785-a-A-21(5'→3')GTCTCGTGGGCTCGGAGATGTGTATAAGAGACAGGACTACHVGGGTATCTAATCC. To each 5 µl of extracted template DNA we added 12.5 µL of a NEBNext Ultra QII 5 Master Mix (New England Biolabs. Frankfurt, Germany), 1.25 µL of each forward and reverse primers (10 pmol/µl) and 5µl of DEPC water. A first PCR was performed as follows: 30s at 98°C, followed by 30 amplification cycles of 10s at 98°C, 30s at 60°C, and 30s at 72°C, and finally 2 min at 72°C. A second PCR was conducted with dual indexing Illumina adaptors from the Nextera XT Index Kit v2 Set B (Illumina, California, USA). One µl of purified amplicon sample (10 ng) was mixed with 2.5 µl of each Illumina index, 12.5 µl of the NEBNext Ultra QII 5 Master Mix and 6.5 µl of DEPC water. The amplification setting was as following: 30s at 98°C, followed by 8 amplification cycles of 10s at 98°C, 30s at 55°C, and 30s at 72°C, with a final heating step at 72°C for 2 min (6). Indexed PCR products were purified using CleanNGS beads (CleanNA, Netherlands) and analysed with Agilent DNA 7500 Chip (Agilent, Waldbronn, Germany). DNA concentrations were measured using QuantiFluor dsDNA System (Promega. Madison, USA) using a Quantus fluorometer (Promega. Madison, USA). The composite pool was prepared by combining 4 nM

of purified DNA samples ensuring equal representations of barcoded libraries. The final pool was sequenced on an Illumina MiSeq platform with a PE300 v3 cartridge generating up to 25 million of 2×300 bp reads. Control samples did not produce measurable amplicons and therefore have been added to the pool in equal volumes instead.

16S downstream sequence processing and statistical analyses

The obtained V3-V4 amplicon reads were analysed following the UPARSE method (7) as implemented in the online IMNGS platform (8). Briefly, primer and barcode sequences were trimmed from each read, and sequences shorter than 200bp, low-quality and chimeras were discarded. The cleaned sequences were clustered into operational taxonomic units (OTUs) based on a similarity cut-off of 97% and taxonomically classified with the RDB classifier (9). Rarefaction curves were additionally generated to assess sampling saturation. Downstream analysis including diversity, taxonomy binning, serial group comparison and correlations were performed using R scripts available in the Rhea pipeline (10). These scripts rely on the R packages ade4, GUniFrac, phangorn, Hmisc, corrplot, plotrix, PerformanceAnalytics, reshape, ggplot2, gridExtra, grid, ggrepel, gtable and their dependencies (R software 4.0.2).

A batch effect correction has been performed using the Combat (11) tool to account for the major confounders, namely the sampling site, the gender and age of participants, being the major source of batch effect according to our PVCA analysis (Supplementary Figure S3C). To correct the age effect, the participants have been categorized into four age groups as follows: below 25, between 25 and 40, between 40 and 65, and over 65. In our analysis, we used a prevalence cut-off of 0.25% and abundance cut-off of 0.5. Based on these cut-offs, 154 OTUs were removed from a total number of 387. Taxa below the abundance cut-off were zeroed and skipped in the statistical analysis to not inflate the significance. As shown in Table 1, a number of patients received therapy (retinoids or low dose naltrexone) during microbiome samplings, and thus we have assessed their impact on the microbiome of the IDS and NIDS skin. To validate the obtained α-diversity data, we calculated the effective richness that takes into account the effect of sequencing depth and spurious taxa (12). Visualization of distance matrices in a space of two dimensions was performed by PCoA (principal coordinate analysis) plots of β-diversity. A PERMANOVA test (vegan::adonis) was achieved to determine if the separation between groups was significant, as a whole and in pairs. The non-parametric Kruskal Wallis Rank Sum test was used for multiple group comparisons. The Mann Whitney test was performed when only two groups were compared, using paired statistics for IDS vs NIDS comparison and unpaired statistics for Control vs IDS and Control vs NIDS comparisons. Multiple test corrections were performed with the Benjamini and Hochberg procedure and corrected *P* values of less than 0.05 considered as significant.

A correlation analysis has been performed using the Pearson's coefficient to investigate the interactions between OTUs or between OTUs and metavariables. Taking into consideration that in

addition to inflamed skin we also sampled non-inflamed DD skin, the number of samples is thus twice as big as for the control group. To correct for that, we considered twice the control group for the analysis in order to balance the patient vs control comparisons. Due to the compositional nature and high sparsity of the taxonomic variables, for correlation analysis of taxonomic data with metavariables, we performed a centred log-ratio transformation to remove the compositional constrains from the taxonomic data. In addition, taxa with a relative abundance value of zero (taxonomic zeros) were considered as missing data and excluded from the correlation's calculation. Following this transformation, the table was centred and scaled, to adjust for differences in the offset and fold changes respectively, and the Pearson correlation for all pairs was calculated. The significance was assessed before and after FDR correction. The linear discriminant analysis effect size (LEFSe) (13) was performed to search for a linear combination of variables (OTUs) that best separates the groups. It employs Kruskal-Wallis rank sum test to detect OTUs with significant differential abundances between groups, the pairwise Wilcoxon test between sub-groups, followed by a linear discriminant analysis (LDA) applied on taxa that meet the significance threshold to estimate their effect size. The OTUs that pass the pairwise Wilcoxon test are considered as potential biomarkers and are ranked according to their LDA scores. An additional heatmap shows the abundance of the key microbial taxa across the different groups of skin phenotype and sampling areas. Although based on relative abundances values, the displayed heatmap rather shows the categories of increase in relative abundance (High, low). When the calculated LDA score is high for a given taxon, the heat map will indicate with which group this taxon is associated if it displays a high abundance.

We constructed furthermore an interaction network using the SparCC (14) (Sparse Correlations for Compositional data) method to define associations between the different taxa. This approach uses a log ratio transformation and performs iterations to identify taxa pairs that are outliers to background correlations. Each node represents a taxon and its size is proportional to the number of connections. Taxa are only connected if the correlation meets a p-value cut-off of 0.05 and a correlation coefficient of 0.3. Considering that taxonomic variables usually differ from metavariables by their compositional nature and high sparsity a centred log-ratio transformation was used to remove the compositional constrains from the taxonomic variables. Key correlations with the Staphylococcus genus are highlighted in the figure with blue and red lines respectively representing negative and positive correlations. The calculated correlations were corrected for false discovery rates (FDR).

RNAseq library preparation and downstream analysis

RNASeq libraries were generated from purified RNA samples using the TruSeq Stranded Total RNA Kit (Illumina) according to manufacturer's protocol. Obtained libraries were sequenced on an

Illumina HiSeq4000 as paired-end with a read length of 2x 150 bp and an average output of 40 Mio reads per sample and end. STAR aligner was used to perform sequence alignment with the human reference genome hg38 (15). Short and low quality reads were discarded and the obtained clean reads were processed using the DESEq2 package for differential gene expression analysis (FCH ≥ 1.5, adj.p< 0.05 and FDR< 0.05) (16, 17). Batch effects were analysed using combat (11) implemented in the sva package to correct for effects of sequencing, age, gender and skin sampling areas. Reads were normalized to counts per million (CPM) and only genes with levels above 0.5 CPM retained. Counts data were log transformed for clustering and principal component analysis (PCA) analysis using EdgeR (18). We constructed a network for the most DEGs between IDS and NIDS skin transcriptomes using the STRINGS platform (19) and visualized it on Cytoscape 3 (20, 21). Gene clusters were identified with ClusterViz using the MCODE algorithm (Molecular complex detection). The number of gene clusters was determined using the elbow method (22). Pathway analysis was performed using the gene ontology analysis (GO) approach including a comparison of different data bases as Biological process, KEGG (Kyoto Encyclopedia of genes and genomes), Biocarta and reactome (23). Gene set enrichment analysis (GSEA) has been performed using the GSEA 4.1.0 platform through which DD transcriptome data were analysed for enrichment in different gene set clusters implemented in databases including KEGG, GOBP (Gene ontology for biological process), HP (Human phenotype), PID (Pathway interaction database). GSEA is a computational approach that determines whether a defined set of genes shows statistically significant differences between two groups. It calculates an enrichment score (ES) by walking down the ranked-ordered list of genes, increasing a running-sum statistic when a gene is present in the gene set and decreasing it if absent. A positive and negative ES respectively indicate an enrichment at the top or the bottom of the ranked gene list. The Th17 gene set cluster was selected from the study of Hu et 2017 (24) and the psoriasis gene set cluster from the SUAREZ FARINAS psoriasis cohort (25). Correlations between abundant taxa and DD transcriptome signatures were analysed on R, corrected for multiple testing using the Benjamini−Hochberg method then displayed on Cytoscape 3.

Histological analysis

Biopsies collected from NIDS and IDS skin were fixed in 4% paraformaldehyde for 24 hours at room temperature, then embedded in paraffin and sectioned at 4 µm. Hematoxylin and eosin staining was performed using the automated tissue stainer COT 20 (MEDITE, Burgdorf, Germany). The immunohistochemical staining (IHC) was performed on 4 µm sections of formalin-fixed paraffin-embedded (FFPE) specimens using the immunostainer BOND-MAX (Zytomed Systems, Berlin, Germany). Briefly, the FFPE sections were incubated with the human anti-CD4 antibody (1:2,

monoclonal rabbit anti-CD4 IgG, (SP35), Zytomed Systems, Berlin, Germany) or the anti-IL-17A antibody (1:20, polyclonal goat IgG (AF-317-NA), bio-techne, Wiesbaden, Germany), then detected using the BOND Polymer Refine Red Detection (Leica Biosystems, Newcastle upon Tyne, UK). Representative images were taken by an experienced comparative pathologist and typical disease features of acantholysis, corps ronds, grains and cellular infiltrates were examined.

Artwork

Figure 1c and Supplementary Figure 1b were created with BioRender.com.

References

1. Severity scoring of atopic dermatitis: the SCORAD index. Consensus Report of the European Task Force on Atopic Dermatitis. *Dermatology.* 1993;186(1):23-31.
2. Troccaz M, Gaïa N, Beccucci S, Schrenzel J, Cayeux I, Starkenmann C, et al. Mapping axillary microbiota responsible for body odours using a culture-independent approach. *Microbiome.* 2015;3(1):3.
3. Peterson J, Garges S, Giovanni M, McInnes P, Wang L, Schloss JA, et al. The NIH Human Microbiome Project. *Genome Res.* 2009;19(12):2317-23.
4. Zeeuwen PL, Boekhorst J, van den Bogaard EH, de Koning HD, van de Kerkhof PM, Saulnier DM, et al. Microbiome dynamics of human epidermis following skin barrier disruption. *Genome Biol.* 2012;13(11):R101.
5. Amar Y, Lagkouvardos I, Silva RL, Ishola OA, Foesel BU, Kublik S, et al. Pre-digest of unprotected DNA by Benzonase improves the representation of living skin bacteria and efficiently depletes host DNA. *Microbiome.* 2021;9(1):123.
6. Klindworth A, Pruesse E, Schweer T, Peplies J, Quast C, Horn M, et al. Evaluation of general 16S ribosomal RNA gene PCR primers for classical and next-generation sequencing-based diversity studies. *Nucleic Acids Res.* 2013;41(1):e1.
7. Edgar RC. UPARSE: highly accurate OTU sequences from microbial amplicon reads. *Nat Methods.* 2013;10(10):996-8.
8. Lagkouvardos I, Joseph D, Kapfhammer M, Giritli S, Horn M, Haller D, et al. IMNGS: A comprehensive open resource of processed 16S rRNA microbial profiles for ecology and diversity studies. *Sci Rep.* 2016;6:33721.
9. Wang Q, Garrity GM, Tiedje JM, and Cole JR. Naive Bayesian classifier for rapid assignment of rRNA sequences into the new bacterial taxonomy. *Appl Environ Microbiol.* 2007;73(16):5261-7.
10. Lagkouvardos I, Fischer S, Kumar N, and Clavel T. Rhea: a transparent and modular R pipeline for microbial profiling based on 16S rRNA gene amplicons. *PeerJ.* 2017;5:e2836.
11. Johnson WE, Li C, and Rabinovic A. Adjusting batch effects in microarray expression data using empirical Bayes methods. *Biostatistics.* 2007;8(1):118-27.
12. Reitmeier S, Hitch TCA, Treichel N, Fikas N, Hausmann B, Ramer-Tait AE, et al. Handling of spurious sequences affects the outcome of high-throughput 16S rRNA gene amplicon profiling. *ISME Communications.* 2021;1(1):31.
13. Segata N, Izard J, Waldron L, Gevers D, Miropolsky L, Garrett WS, et al. Metagenomic biomarker discovery and explanation. *Genome Biol.* 2011;12(6):R60.

14. Friedman J, and Alm EJ. Inferring correlation networks from genomic survey data. *PLoS Comput Biol.* 2012;8(9):e1002687.
15. Dobin A, Davis CA, Schlesinger F, Drenkow J, Zaleski C, Jha S, et al. STAR: ultrafast universal RNA-seq aligner. *Bioinformatics.* 2013;29(1):15-21.
16. Anders S, and Huber W. Differential expression analysis for sequence count data. *Genome Biol.* 2010;11(10):R106.
17. Love MI, Huber W, and Anders S. Moderated estimation of fold change and dispersion for RNA-seq data with DESeq2. *Genome Biol.* 2014;15(12):550.
18. Robinson MD, McCarthy DJ, and Smyth GK. edgeR: a Bioconductor package for differential expression analysis of digital gene expression data. *Bioinformatics.* 2010;26(1):139-40.
19. Szklarczyk D, Gable AL, Lyon D, Junge A, Wyder S, Huerta-Cepas J, et al. STRING v11: protein-protein association networks with increased coverage, supporting functional discovery in genome-wide experimental datasets. *Nucleic Acids Res.* 2019;47(D1):D607-d13.
20. Shannon P, Markiel A, Ozier O, Baliga NS, Wang JT, Ramage D, et al. Cytoscape: a software environment for integrated models of biomolecular interaction networks. *Genome Res.* 2003;13(11):2498-504.
21. Su G, Morris JH, Demchak B, and Bader GD. Biological network exploration with Cytoscape 3. *Curr Protoc Bioinformatics.* 2014;47:8.13.1-24.
22. Nanjundan S, Sankaran S, Arjun CR, and Paavai Anand G. 2019:arXiv:1912.00643.
23. Subramanian A, Tamayo P, Mootha VK, Mukherjee S, Ebert BL, Gillette MA, et al. Gene set enrichment analysis: a knowledge-based approach for interpreting genome-wide expression profiles. *Proc Natl Acad Sci U S A.* 2005;102(43):15545-50.
24. Hu D, Notarbartolo S, Croonenborghs T, Patel B, Cialic R, Yang TH, et al. Transcriptional signature of human pro-inflammatory T(H)17 cells identifies reduced IL10 gene expression in multiple sclerosis. *Nat Commun.* 2017;8(1):1600.
25. Suárez-Fariñas M, Lowes MA, Zaba LC, and Krueger JG. Evaluation of the psoriasis transcriptome across different studies by gene set enrichment analysis (GSEA). *PLoS One.* 2010;5(4):e10247.
